# Anti-adipogenic signals at the onset of obesity-related inflammation in white adipose tissue

**DOI:** 10.1007/s00018-020-03485-z

**Published:** 2020-03-11

**Authors:** Tiziana Caputo, Van Du T. Tran, Nasim Bararpour, Carine Winkler, Gabriela Aguileta, Khanh Bao Trang, Greta M. P. Giordano Attianese, Anne Wilson, Aurelien Thomas, Marco Pagni, Nicolas Guex, Béatrice Desvergne, Federica Gilardi

**Affiliations:** 1 Center for Integrative Genomics, University of Lausanne, Lausanne, Switzerland; 2 Vital-IT Group, SIB Swiss Institute of Bioinformatics, Lausanne, Switzerland; 3 Unit of Forensic Toxicology and Chemistry, CURML, Lausanne University Hospital, Geneva University Hospitals, Lausanne, Switzerland; 4 Faculty Unit of Toxicology, Faculty of Biology and Medicine, CURML, Lausanne University Hospital, University of Lausanne, Lausanne, Switzerland; 5 Department of Oncology, University of Lausanne, Epalinges, Switzerland; 6 Bioinformatics Competence Center, University of Lausanne, Lausanne, Switzerland

**Keywords:** System biology, Adipose tissue, Metaflammation, Epigenetics, Adipogenesis, Angiogenesis, Adipocyte precursors, Transcriptomics, Genome-scale metabolic network

## Abstract

Chronic inflammation that affects primarily metabolic organs, such as white adipose tissue (WAT), is considered as a major cause of human obesity-associated co-morbidities. However, the molecular mechanisms initiating this inflammation in WAT are poorly understood. By combining transcriptomics, ChIP-seq and modeling approaches, we studied the global early and late responses to a high-fat diet (HFD) in visceral (vWAT) and subcutaneous (scWAT) AT, the first being more prone to obesity-induced inflammation. HFD rapidly triggers proliferation of adipocyte precursors within vWAT. However, concomitant antiadipogenic signals limit vWAT hyperplastic expansion by interfering with the differentiation of proliferating adipocyte precursors. Conversely, in scWAT, residing beige adipocytes lose their oxidizing properties and allow storage of excessive fatty acids. This phase is followed by tissue hyperplastic growth and increased angiogenic signals, which further enable scWAT expansion without generating inflammation. Our data indicate that scWAT and vWAT differential ability to modulate adipocyte number and differentiation in response to obesogenic stimuli has a crucial impact on the different susceptibility to obesity-related inflammation of these adipose tissue depots.

**Electronic supplementary material:**

The online version of this article (10.1007/s00018-020-03485-z) contains supplementary material, which is available to authorized users.

## Introduction

Human obesity has become a global epidemic, causing high mortality especially due to the many associated co-morbidities. Although carrying a large amount of fat is not necessarily detrimental, human obesity is accompanied by systemic chronic inflammation, which plays an important role in the pathogenesis of obesity-related insulin resistance [11].

White adipose tissue (WAT) is the main location for lipid storage and expands in response to a high-fat diet (HFD) or over-nutrition. WAT expansion occurs by either increasing the size of individual adipocytes (hypertrophy), or by recruiting new adipocytes (hyperplasia). In the latter, since mature adipocytes are post-mitotic, the newly generated adipocytes derive from adipocyte progenitors (APs), which are located in the stromal vascular fraction (SVF) of the WAT. Adipocyte differentiation is a two-phase process. The determination phase, from AP to pre-adipocyte, is followed by terminal differentiation, where committed pre-adipocytes acquire the characteristics of mature adipocytes [35]. This two-step differentiation process requires a temporally highly regulated transcriptional network that has been extensively studied over the past three decades [19, 44].

When considering the impact on the development of metabolic disorders, two main types of WAT have been identified: subcutaneous WAT (scWAT) and visceral WAT (vWAT), which have distinct properties in terms of cell composition, gene expression, developmental lineage, and adipokine secretion [16, 28].

scWAT hosts not only white adipocytes, but also clusters of “beige” adipocytes which, despite being a distinct cell type compared to the classical brown adipocytes [40], share the same thermogenic capacity due to expression of the uncoupling protein 1 (UCP1). This brown AT-specific protein has the function of dissipating the proton gradient produced by the respiratory chain, therefore reducing the efficiency of ATP synthesis and dissipating energy in the form of heat.

The difference between vWAT and scWAT is also important with respect to the response to obesity-induced inflammation. vWAT is more prone to develop inflammation in the context of over-nutrition with recruitment of a number of immune cells [13, 42]. In this tissue, hypertrophic growth of adipocytes is associated with a relative deficiency of vasculature that creates a local imbalance between oxygen supply and consumption, which in turn, leads to increased levels of angiogenic factors and expression of inflammation and endoplasmic reticulum stress-associated genes [12].

While numerous reports describe the mechanisms by which inflammation induces insulin resistance, very little is known about the molecular processes that lead to the onset of inflammation in obesity. We, thus, took advantage of the different behavior of vWAT and scWAT to identify the early specific effects of a HFD on vWAT that may cause the subsequent inflammation. To this purpose, we used a combination of system analyses, comprising RNA-seq and ChIP-seq of major epigenetic marks together with a metabolic modeling strategy, to identify key differences in the responses of vWAT and scWAT to HFD after 1, 8 and 20 weeks. The loss of beige adipocytes in scWAT and the appearance of anti-adipogenic signals in vWAT are two of the major findings that this study further experimentally studied.

## Results

### HFD feeding induces an early modulation of distinct pathways in visceral and subcutaneous adipose tissue

**Fig. 1:**
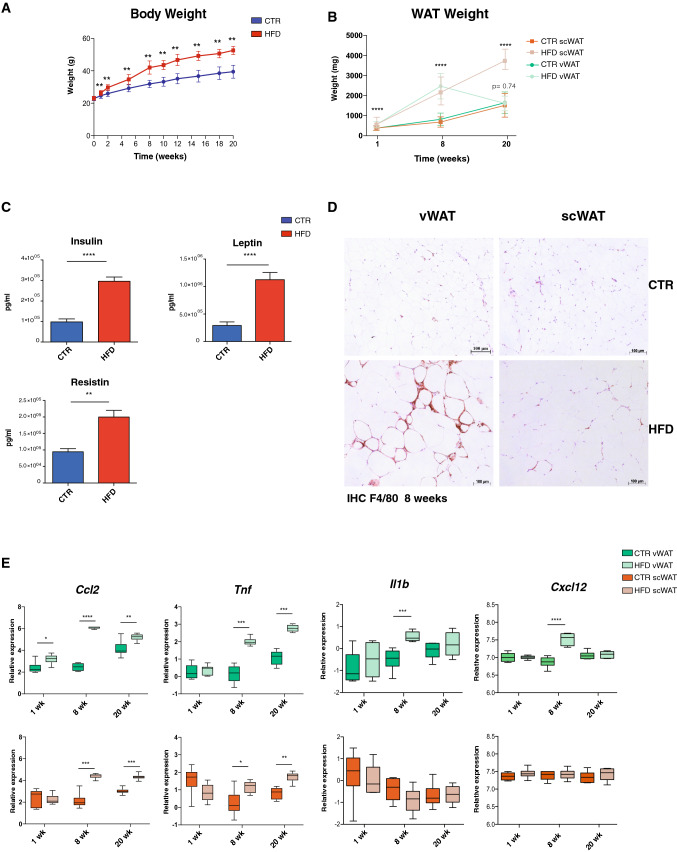
HFD effects on weight and on the inflammatory pattern in mice. **a** Evolution of weight gain during the 20 weeks of HFD treatment. **b** Ex vivo measurements of vWAT and scWAT weight after 1, 8 and 20 weeks of HFD treatment. **c** Measurement of insulin, resistin and leptin circulating levels. **d** Representative images of F4/80 immunohistochemistry in control (top) and 8-week HFD (bottom) in vWAT and scWAT. **e** Log2 gene expression levels of inflammatory marker genes *Ccl2, Tnf*, *Il1b* and *Cxcl12* in vWAT (top) and scWAT (bottom) from controls or HFD-treated mice after 1, 8 and 20 weeks (*n* = 6). Data are represented as mean ± SD. Two-tailed Student’s *t* test was used to calculate the significant changes between control and HFD mice. *(*p* value < 0.05), **(*p* value < 0.01), ***(*p* value < 0.001), ****(*p* value < 0.0001)

To reveal the early events that occur in WAT upon a HFD, and explain its pathological modifications associated with the development of inflammation, we analyzed the transcriptomic changes induced by a HFD in C57Bl6/J male mice after 1, 8 and 20 weeks (Fig. S1A). The mice fed with a HFD showed a significant difference in weight after only 1 week of diet (Fig. 1a), which correlated with an expansion of WAT (Fig. 1b). Interestingly, at 20 weeks, the amount of scWAT was greatly increased, whereas vWAT displayed a loss of weight with no significant differences between the control and HFD group. As expected, mice under a HFD showed increased serum levels of insulin, leptin and resistin (Fig. 1c) and developed inflammation mainly in vWAT at 8 weeks, as shown by the higher number of F4/80^+^ cells and crown-like structures compared to scWAT as well as by an increased expression of inflammatory markers such as *Ccl2, Tnf*, *Il1b* and *Cxcl2* (Fig. 1d, e).

**Fig. 2:**
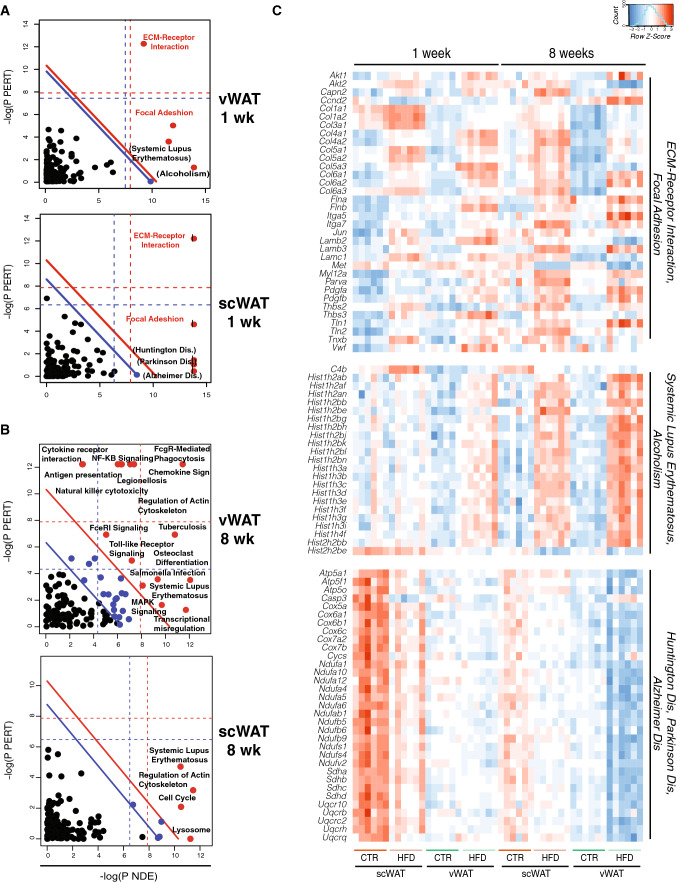
Transcriptomic response to HFD feeding of visceral and subcutaneous adipose tissue is characterized by the modulation of distinct pathways. **a**, **b** Two-dimensional plots illustrating the signaling pathway impact analysis (SPIA) performed on the RNAseq data obtained from vWAT and scWAT after 1 week (**a**) and 8 weeks (**b**) of HFD. The *X*-axis shows the over-representation evidence (P_NDE_), while the *Y*-axis shows the perturbation evidence (P_TERT_). Each pathway is represented by a dot. Pathways above the oblique red line (red dots) are significant at 5% after Bonferroni correction, while those above the oblique blue line are significant at 5% after FDR correction (black blue dots). The vertical and horizontal thresholds represent the same corrections for the two types of evidence considered individually. Pathways with a log(P_NDE_) higher than 15 are marked by an additional small vertical bar. **c** Heatmap of log2 expression value of shared genes in the “ECM–receptor interaction” and “Focal adhesion”, “Systemic lupus erythematosus” and “Alcoholism” and “Parkinson dis.”, “Huntington dis.” and “Alzheimer dis.” pathways at 1 and 8 weeks in both vWAT and scWAT

To get an overview of gene expression changes upon a HFD, we performed RNA-seq in both vWAT and scWAT at the different time points and applied the pathway analysis known as signaling pathway impact analysis (SPIA [43]) to the results. Two main pathways emerged as commonly deregulated by 1 week of HFD in both vWAT and scWAT, the ECM receptor interaction and focal adhesion pathways, reflected by the increased expression of genes coding for membrane proteins mainly related to collagen genes (Fig. 2a, c and Table S1).

In contrast, some pathways were differentially regulated exclusively in only one of the two tissues. Systemic Lupus Erythematosus (SLE) and Alcoholism pathways were deregulated only in vWAT. More particularly, 27 histone genes common to both these pathways and coding for all the canonical nucleosomal histones (H2A, H2B, H3, H4), showed increased expression in vWAT under the HFD condition (Fig. 2c and Table S2).

Conversely, in scWAT, three other pathways linked to neurodegenerative diseases appear deregulated at 1 week, i.e., Huntington, Parkinson and Alzheimer diseases. Interestingly, the group of genes shared between these three pathways, and down-regulated under the HFD condition only in scWAT, mainly belong to the electron transport chain (ETC) and mitochondrial function (Fig. 2c and Table S3).

At 8 weeks of HFD treatment, consistently with our and previous observations (Fig. 1d, e; [13, 42]), the deregulated pathways appearing in vWAT showed a clear inflammatory response, with a number of pathways linked to inflammation, including NF-KB and TNFα signaling (Fig. 2b). In contrast, no pathways linked to inflammation were present in scWAT after 8 weeks of diet.

Finally at 20 weeks, whereas vWAT remained highly affected by the inflammatory response (Fig. S2A), scWAT displayed a deregulation of SLE and Cell cycle pathways, which is maintained from 8 weeks onwards (Table S4A). As for vWAT at 1 week of HFD, the upregulated genes in SLE were mostly histone genes (Table S4B). In addition, two significant pathways related to inflammation appeared in scWAT (“Chemokine signaling pathways” and “Leukocyte transendothelial migration” pathways).

Taken together, these data show that vWAT and scWAT have, in the early phase, a distinct response to the diet, involving histone genes and genes linked to mitochondrial activity in vWAT and scWAT, respectively, followed by the onset of a strong inflammatory response only in vWAT.

### scWAT undergoes loss of beige adipocytes in the early phase of overnutrition

**Fig. 3:**
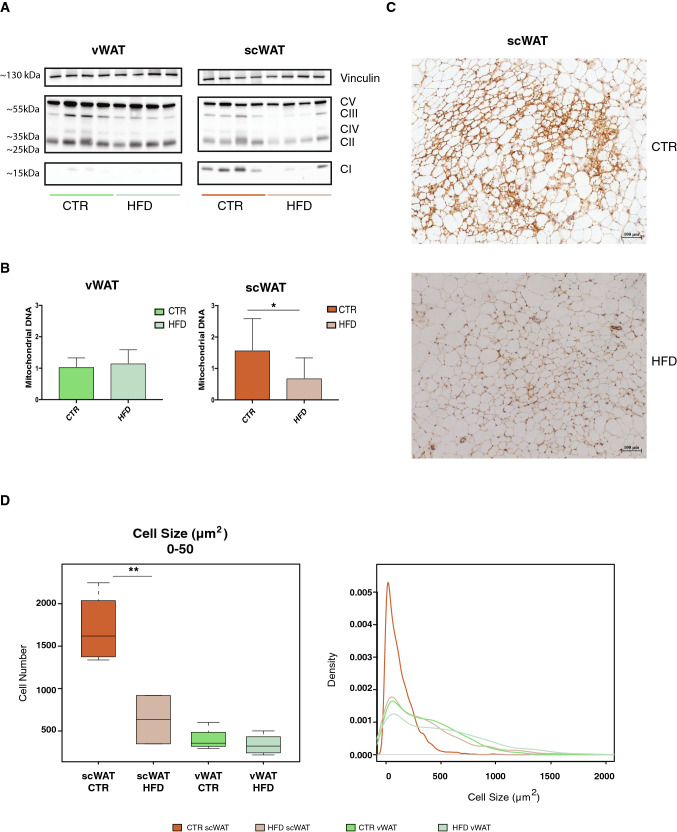
The early response to overnutrition in scWAT is characterized by loss of beige adipocytes. **a** Western blot for total OXPHOS proteins in scWAT from controls and HFD (*n* = 4) after 1 week. Vinculin is used as a loading control. **b** Mitochondrial DNA quantification. The *y*-axis represents the ratio between expression of a mitochondrial 16S and a genomic gene (ln11). **c** Representative images of UCP1 immunohistochemistry in control (top) and 1-week HFD (bottom). **d**)Cell size distribution calculated on sections of vWAT and scWAT from controls and 1-week HFD-treated mice (*n* = 5). Left panel: quantification of cells ranging from 0 to 50 µm^2^. Right panel: the *y*-axis shows the probability density function for the kernel density estimation and the *x*-axis the cell size, ranging from 0 to 2000 µm^2^ (*n* = 5). Data are represented as mean ± SD. Significance between the indicated groups was calculated using a two-tailed Student’s *t* test. *(*p* value < 0.05), **(*p* value < 0.01)

As shown in Fig. 2, the early transcriptional changes occurring specifically in scWAT upon HFD are linked to the reduced expression of ETC-related genes. To validate this observation, we looked at the levels of some of the corresponding proteins by Western blot. Indeed, the decrease of complexes I, III, IV occurs also at the protein level in scWAT upon HFD (Fig. 3a). This decrease in mitochondrial proteins correlates with an overall reduction in mitochondrial mass, as supported by the decrease in mitochondrial DNA, which gives an estimation of mitochondrial abundance (Fig. 3b). Thus, the observed down-regulation of mitochondrial respiration can be due to a global reduction of mitochondrial number.

scWAT is known to harbor both white and beige adipocytes [30, 40], and, therefore, to have a higher mitochondrial mass compared to visceral fat. To investigate if tissue composition was altered in scWAT upon HFD, we performed histological analysis to quantify the number of beige adipocytes (UCP1^**+**^) in control and HFD-treated mice. As shown in Fig. 3c, scWAT contains many clusters of small UCP1^**+**^ cells with multi-locular lipid droplets, characteristic of beige adipocytes. These features are strongly reduced after 1 week of HFD. The reduction in UCP1^**+**^ cells also correlates with a drop in the number of small cells ranging up to 50 µm^2^ (Fig. 3d), suggesting that the small cells, which disappear upon a HFD, are possibly those contributing to the hypertrophic expansion of white UCP1^**−**^ adipocytes. In contrast, we observe a clear shift in size, with a significant increase in the number of large cells.

These data suggest that beige UCP1^+^ adipocytes in scWAT lose their beige properties and contribute to the expansion of scWAT under a HFD.

### HFD differentially regulates adipocyte progenitor proliferation and differentiation in vWAT and scWAT

The massive increase in histone gene transcription seen already at 1 week in vWAT, and observed later in both vWAT and scWAT at 8 weeks (Fig. 2), could be associated with increased cell proliferation. Consistent with this hypothesis, the expression levels of some known markers of proliferation, such as *Mki67* and *E2f1* (Fig. S2B), were increased only in vWAT after 1 week of HFD, while they were upregulated in both vWAT and scWAT at 8 and 20 weeks.

While proliferation signals observed from 8 weeks on in vWAT might be linked to proliferation of immune cells, which are infiltrating vWAT at this time point (Fig. 1d, e), we explored which cell population is already proliferating upon over-nutrition after 1 week. Interestingly, the expression levels of the *Zfp423* gene, one of the most used markers for AP commitment to preadipocytes, is significantly increased in both tissues at 1 week (Fig. S2B),while it is only increased in scWAT at 8 weeks. Moreover, at 20 weeks *Zfp423* expression is oppositely regulated, being downregulated in vWAT and upregulated and in scWAT. These observations suggest that the proliferating signals observed early in vWAT and at later time points in scWAT could be associated with AP clonal expansion, which represents the first step of their commitment.

**Fig. 4:**
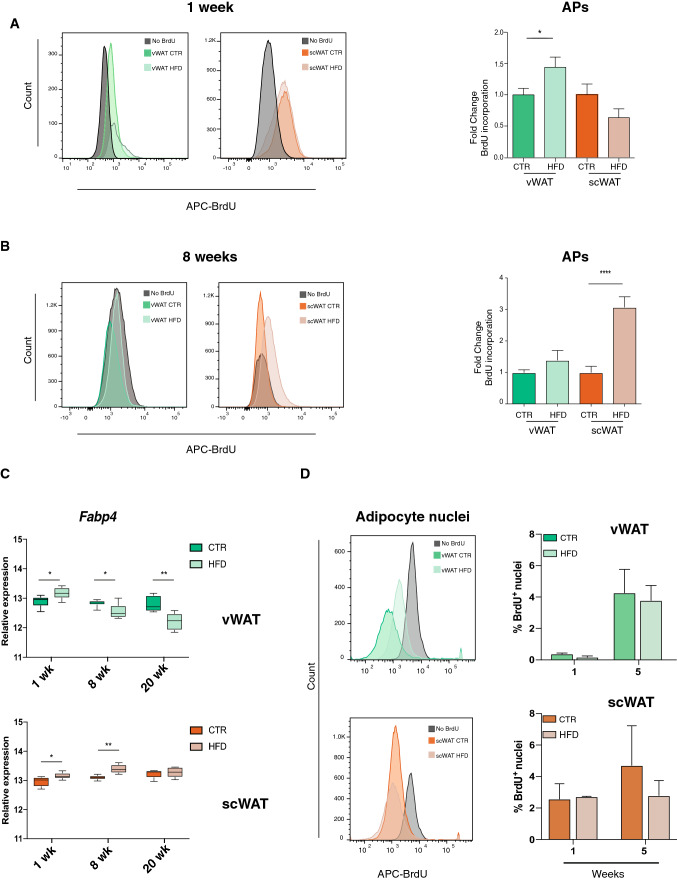
Proliferation of adipocyte progenitors is specific to vWAT in the early response to HFD. **a** Quantification of BrdU incorporation into APs from vWAT or scWAT after 1 week of diet treatment (*n* = 12). **b** Quantification of BrdU incorporation into APs from vWAT or scWAT of male mice that received 1-week BrdU treatment after 7 weeks of diet (*n* = 16). **c** Log2 Gene expression level of the mature adipocyte marker *Fabp4* in vWAT (top) and scWAT (bottom) from control or HFD-treated mice for 1, 8 and 20 weeks (*n* = 6). **d** BrdU incorporation into adipocyte nuclei after treatment after 1 (*n* = 8) or 5 weeks of diet (*n* = 12). **b**, **c** Left panels: schematic representation of BrdU incorporation in control mice not treated with BrdU (gray), and control and HFD-treated mice. The percentage of positive cells was calculated using the same threshold for both vWAT and scWAT. Right panel: the change in BrdU incorporation in AP cells was calculated as fold change over the control mice dissected on the same day. Data are represented as mean ± SD. Significance between the indicated groups was calculated using a two-tailed Student’s *t* test. *(*p* value < 0.05), **(*p* value < 0.01), ****(*p* value < 0.0001)

To confirm the distinct timing of AP commitment regulation in response to a HFD in the different AT depots, we exposed mice to bromodeoxyuridine (BrdU) either during the first week or the seventh week of diet (Fig. S1b, c). FACS analysis performed using the classical markers for AP identification (CD31^−^: CD45^−^: CD29^+^: CD34^+^: Sca-1^+^) showed increased BrdU incorporation only in vWAT during the first week of HFD treatment (Fig. 4a), indicating a rapid increase of AP proliferation in this tissue. In contrast, mice exposed to BrdU later in the HFD treatment, revealed a strong burst of AP proliferation only in scWAT (Fig. 4b), confirming the hyperplastic growth of this tissue after 8 weeks of diet.

Taken together, these results suggest that the increase in histone gene transcription seen in vWAT by pathway analysis is associated with AP commitment at the early stage of over nutrition. Conversely, in scWAT, the increased transcription of histone genes, likely associated with AP commitment, is only observed upon 8 and 20 weeks of HFD treatment (Figs. 2b, S2A).

For a healthy expansion of adipose tissue, AP commitment must be followed by terminal differentiation. We, thus, explored the influence of HFD on adipocyte differentiation in the two adipose tissue depots by looking, first, at the expression of known markers of mature adipocytes, such as PPARγ and Fabp4. As shown in Fig. S2C, *Pparg* expression was reduced by HFD in both vWAT and scWAT. In contrast, *Fabp4* is upregulated by HFD treatment in both tissues at 1 week (Fig. 4c), while it is oppositely regulated at 8 weeks, with increased levels in scWAT and reduced expression in vWAT. Its levels are even more decreased in vWAT at 20 weeks, in parallel to the shrinking of this tissue (Fig. 1b).

Such expression pattern argues against a significant increase of adipocyte differentiation in response to HFD in vWAT. To further investigate this aspect, we treated mice with BrdU during the first week of HFD and the percentage of BrdU+ nuclei in the mature adipocyte fraction was measured after 1 and 5 weeks. As expected, no BrdU-positive nuclei in the mature adipocyte fraction could be seen at 1 week in both tissues (Fig. 4d). However, a general increase in BrdU-positive nuclei was seen after 5 weeks, especially in vWAT, but with no significant differences between control and HFD groups. Thus, although HFD rapidly induces AP clonal expansion in vWAT (Fig. 4a), this is not followed by a proportional increase of adipocyte differentiation, suggesting that the maturation of these committed cells cannot be properly completed.

### HFD feeding induces a different acetylation profile in scWAT and vWAT in genomic regions linked to lipid metabolism and cell fate

**Fig. 5:**
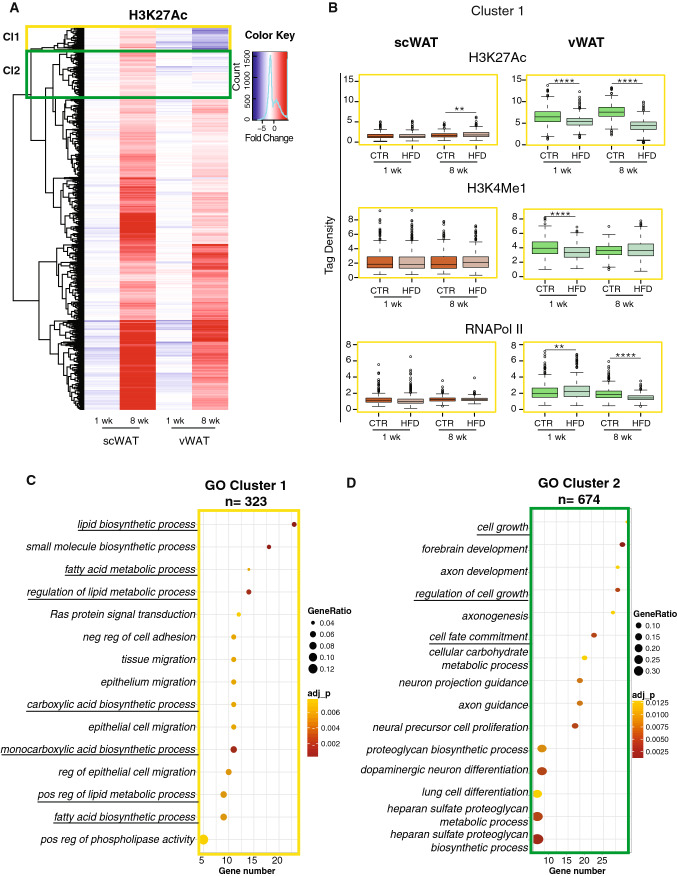
scWAT and vWAT show distinct acetylation profiles in genomic regions linked to lipid metabolism and cell fate upon HFD feeding. **a** ChIP-seq cluster analysis. Hierarchical cluster analysis and heatmap showing the Log fold change (LogFC) of H3K27Ac on 5984 genomic regions, selected based on the significant alteration of their H3K27Ac signals when comparing control and HFD vWAT at 8 weeks. The yellow and the green rectangles highlight the two clusters of interest. **b** Box plot of average tag density on the 359 regions in cluster 1 (yellow frame) for RNAPol II, H3K4me1 and H3K27Ac. Significance between the indicated groups was calculated using a two-tailed Student’s *t* test. *(*p* value < 0.05), **(*p* value < 0.01), ****(*p* value < 0.0001). **c**, **d** Pathway enrichment analysis performed on genes annotated in clusters 1 and 2, respectively. In each panel, the *x*-axis shows the number of genes found in the cluster belonging to the pathway. The dots are colored based on the significance of each pathway and the size of the dots represents the ratio between the number of genes in the cluster and in the pathway

To explore the molecular mechanisms underlying the distinct temporal regulation of AP proliferation and differentiation in vWAT and scWAT in response to the diet, we looked at epigenetic changes affecting transcription. We focused on the ChIP-seq profile of two enhancer markers, H3K27 acetylation (active enhancers), and H3K4 monomethylation, together with that of RNA Pol II recruitment. Retrieving all genomic regions with significant changes in H3K27Ac signals in HFD mice compared to controls (*p* value < 0.05), either in vWAT or in scWAT at 8 weeks, identified a total of 5984 regions. The tag density of these regions was also analyzed in 1-week samples to identify early changes possibly responsible for the onset of inflammation in vWAT only. While most of the tested regions respond similarly to the HFD in scWAT and vWAT, the hierarchical clustering highlighted two interesting clusters (yellow and green square) with a distinct pattern in both tissues (Fig. 5a).

The regions of the first cluster (359 regions) were characterized by increased H3K27Ac in scWAT upon HFD, as opposed to reduced acetylation levels in vWAT. This behavior was already present at 1 week and was more marked after 8 weeks of diet. The second cluster (711 regions) presented a similar acetylation profile but the differences were less marked between 1 and 8 weeks in vWAT. Of note, the total tag density obtained for H3K27Ac and H3K4me1 on the regions of cluster 1 and 2 is very different between vWAT and scWAT under control conditions, with a much lower average tag density in scWAT (Figs. 5b, S3A). This suggests that these enhancers, which are differentiating the response to a HFD in the two depots, are physiologically more accessible for transcription in vWAT than in scWAT. Furthermore, in vWAT, we observed significant changes in RNAPol II recruitment, with an average tag density increased after 1 week of HFD, but reduced after 8 weeks. In parallel, H3K4me1 levels are significantly reduced at 1 week but unchanged at 8 weeks, suggesting that changes in this marker are happening mainly in the early phase of overfeeding. Finally, scWAT is oppositely regulated compared to vWAT only at the level of H3K27Ac tag density at 8 weeks; while, H3K4me1 and RNAPol II are not statistically different (Fig. 5b). Thus, the distinct pattern between vWAT and scWAT mainly stems from the level of H3K27Ac after 8 weeks in the regions of both cluster 1 and cluster 2 (Figs. 5b, S3A).

To gain insights into the putative biological functions of these chromatin regions, we annotated them to the closest gene from their center. This step provided a list of 323 genes from the first cluster and 674 genes from the second one. Gene Ontology annotations of the genes in the first cluster gave a strong enrichment of pathways linked to fatty acid metabolism (Fig. 5c). This was due to the presence of genes like *Ppara, Hacd2, Psapl1, Scd4, Elovl6, Gpat2, Acsf2, Pla2g5, Alox12, Pck1, Degs1, Acsbg1*, which are associated with sphingolipid and long-chain fatty acid metabolism. This cluster also contains developmental genes (*Nrp2, Irx4, Mfng, Tshz2, Shroom3, Nat8f2, Ackr3, Celsr1, Ambra1, Fzd4, Sufu, Farp1, Slit3, Wnt4, Hoxa3, Slc26a8, Smoc1, Asb1, Etl4, Fgf1, Sik1, Ihh, Pitx2, Bmp8a, Zfp423*) and some genes belonging to the Wnt pathway (*Wnt4, Rspo1, Fzd4, Mmp7, Camk2b, Axin2*). Interestingly, the second cluster is also enriched in terms of cell commitment and regulation of cell growth (Fig. 5d). Thus, the decreased H3K27Ac of these regions in vWAT but not in scWAT suggests that, upon HFD treatment, vWAT undergoes a reduction in fatty acid metabolism and, in line with our previous observations, a perturbation of cell growth and cell differentiation.

**Fig. 6:**
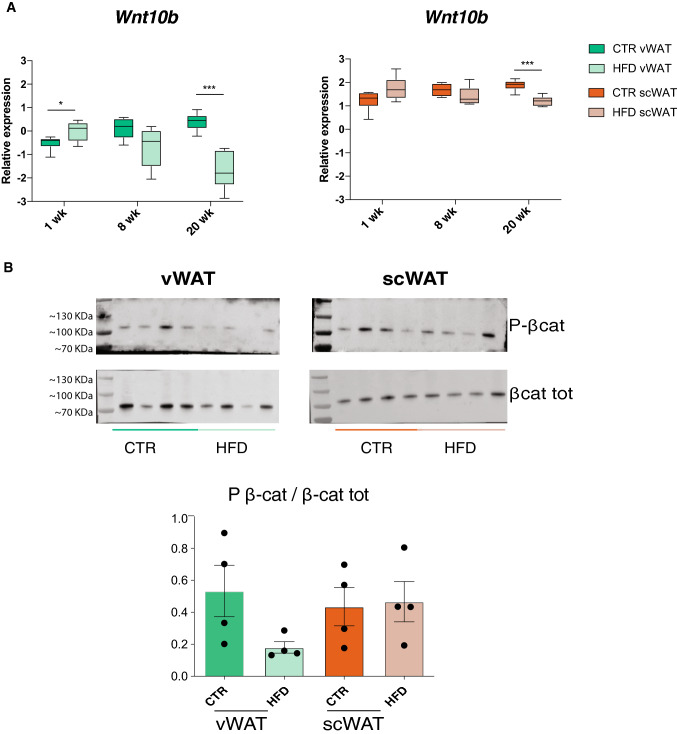
vWAT displays impaired AP differentiation in the late response to HFD. **a** *Wnt10b* gene expression levels in vWAT (top) and scWAT (bottom) at 1, 8 and 20 weeks of diet treatment. Significance between the indicated groups was calculated using a two-tailed Student’s *t* test.*(*p* value < 0.05), ***(*p* value < 0.001). **b** Western blot for phosphorylated and total β-catenin in vWAT after 1-week HFD (*n* = 4), with relative quantification as a ratio between phosphorylated and total β-catenin (bottom). *p < 0.05 for tissue–diet interaction was calculated using 2way ANOVA. Data are represented as mean ± SD

The presence of many genes of the Wnt pathway in both cluster 1 (*Wnt4, Rspo1, Fzd4, Mmp7, Camk2b, Axin2*) and cluster 2 (*Usp34, Nle1, Prickle1, Gsk3b, Tcf7l2, Src, Nfkb1, Dlx5, Mitf, Zfp703, Tnks, Ctnnb1, Smad3)* was particularly interesting as this signaling pathway is known to play a central role in adipocyte differentiation [3, 36]. We, thus, measured the expression of different Wnt genes, which have distinct properties with respect to adipogenesis. Interestingly, many Wnt genes are already differentially expressed in vWAT and scWAT in control mice, with decreased *Wnt2* and *Wnt10b* and increased *Wnt2b*, *Wnt5b* and *Wnt7b*, in vWAT compared to scWAT (Fig. S3B). This suggests that the Wnt pathway and its action might be intrinsically different in the two tissues. Knowing that defects in adipogenesis can have an impact on the generation of hypertrophic adipocytes, we hypothesized a possible involvement of Wnt as a crucial player in the different response of vWAT and scWAT to a HFD. Consistent with this hypothesis, the expression of *Wnt10b*, which is a well-known negative regulator of adipogenesis [3, 4], increases in vWAT after 1 week of diet (Fig. 6a). This up-regulation is accompanied by reduced phosphorylation of β-catenin, further suggesting that the canonical Wnt pathway was likely activated only in vWAT after 1 week of HFD (Fig. 6b). Unphosphorylated β-catenin is less targeted for proteasomal degradation and, thus, free to access the nucleus and activate transcription of anti-adipogenic genes [21, 27].

These results suggest that Wnt10b and activation of the canonical Wnt/β-catenin pathway may be important for the early distinct responses of vWAT and scWAT to a HFD. Altogether, the transcriptomic changes occurring in vWAT may sustain a transient induction of AP commitment in vWAT concomitantly with impairment of the differentiation capacity of committed APs. Conversely, in scWAT, AP commitment is associated with increased expression of markers of mature adipocytes, meaning that no block of differentiation is present in this tissue.

### Opposite modulation of the angiogenic capacity of vWAT and scWAT in response to HFD

The results obtained so far highlighted important biological features, but only a few related to metabolism. We, thus, attempted to investigate further the RNA-seq data with metaboGSE [45], which is a recently published algorithm relying on a genome-scale metabolic network (GSMN) to simulate the cellular metabolism. Albeit limited to metabolic reactions, this method emphasizes the low expressed genes and produces alternative gene set enrichment results, which are not necessarily identified by classical algorithms like GSEA and topGO. Along this approach, the RNA-seq datasets at 1 and 8 weeks were analyzed and 28 pairwise comparisons between the 8 conditions were investigated for 678 GO term-associated gene sets. These gene sets were selected as they were well supported by the GSMN and minimally redundant (see “Methods” details).

The overall analysis with the 28 contrasts showed that the difference between tissues was dominating over the diet and time-point variations (Fig. S4A). In line with the previous observations [46, 50], metaboGSE highlighted the thermogenic capacity of scWAT, confirming the appropriate functionality of the method.

When comparing the diet, a set of biological processes related to cGMP signaling, nitric oxide, hyaluronan biosynthesis, blood pressure regulation, and prostaglandin secretion appeared to be weakly but consistently enriched across the 4 pairwise comparisons. Of note, a dysregulation of the cGMP signaling was already described in adipose tissue from obese patients, in association with inflammation [38].

**Fig. 7:**
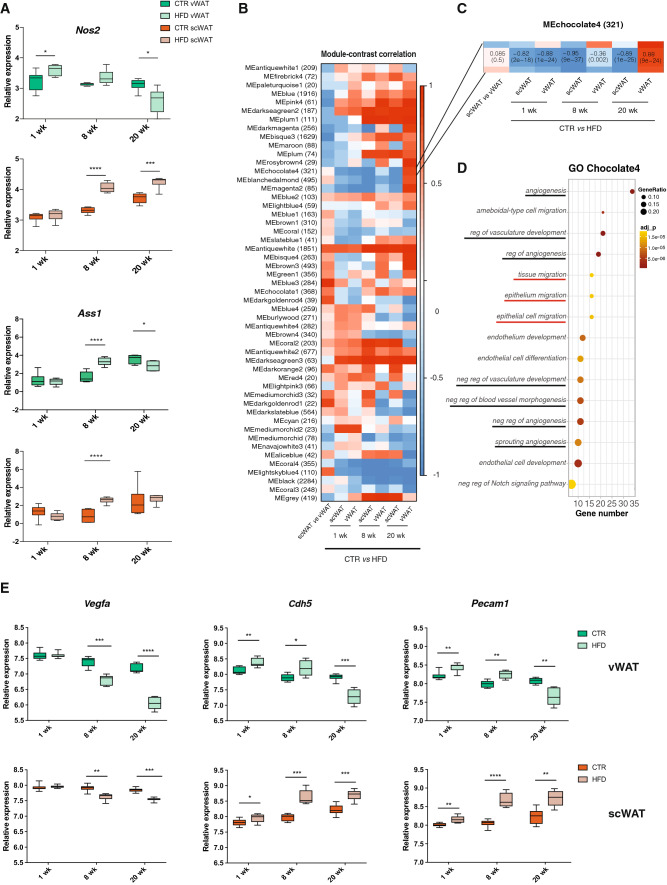
Pathway analysis using WGCNA reveals interesting new genes differentiating vWAT and scWAT responses to HFD. **a** Log2 Gene expression level of *Nos2* and *Ass1* in vWAT and scWAT of controls or HFD-treated mice for 1, 8 and 20 weeks (n = 6). **b** Heatmap of the WGCNA output. Each row represents a module containing the number of genes reported in parenthesis. Each column represents a contrast as indicated below the heatmap, from left to right: (1) tissue differences between CTR vWAT and CTR scWAT, (2) HFD-dependent effects in scWAT after 1 week, (3) HFD-dependent effects in vWAT after 1 week, (4) HFD-dependent effects in scWAT after 8 weeks, (5) HFD-dependent effects in vWAT after 8 weeks, (6) HFD-dependent effects in scWAT after 20 weeks, (7) HFD-dependent effects in vWAT after 20 weeks. **c** Zoom on the Chocolate4 module. **d** Pathway enrichment analysis performed on the genes contained in the Chocolate4 module (*n* = 321 genes). The dots are colored based on the significance of each pathway and the size represents the ratio between the number of genes in the cluster and in the pathway. Black underlined pathways are related to angiogenic processes, while the red ones are in common with pathways in Fig. 5. **e** Log2 Gene expression levels of the angiogenesis and epithelial cell-related genes *Vegfa, Cdh5* and *Pecam1* in vWAT (top) and scWAT (bottom) of controls or HFD-treated mice after 1, 8 and 20 weeks (*n* = 6). For **a**, **e** data are represented as mean ± SD. Significance between the indicated groups was calculated using a two-tailed Student’s *t* test. *(*p* value < 0.05), **(*p* value < 0.01), ***(*p* value < 0.001), ****(*p* value < 0.0001)

Genes belonging to these 5 main biological processes were systematically removed from the model to test their effect on the GSMN and to identify those with the highest impact (see “Methods”). The simulation highlighted the following five genes as most impactful: Nitric oxide synthase *Nos2*, Argininosuccinate synthase *Ass1*, Hexokinase-3 *Hk3,* GTP cyclohydrolase 1 *Gch1*, and 4-aminobutyrate aminotransferase *Abat* (see “Methods” details and Figs. S4B, S5). Among them, *Ass1* and *Nos2* are involved in the same metabolic pathway, with ASS1 being a key enzyme in the metabolism of arginine, providing l-arginine as substrate for NOS proteins. *Nos2* and *Ass1* were significantly upregulated in vWAT at 1 and 8 weeks, respectively; while, they were reduced at later time point (20 weeks). Instead, in scWAT, both genes were unchanged after 1 week, but increased at 8 and 20 weeks compared to the controls (Fig. 7a). Such expression profile, particularly that of *Nos2,* was similar to that of adipocyte commitment and differentiation markers (i.e., *Zfp423* and *Fabp4),* when considering the tissue difference in response to the diet. This observation prompted us to search for other genes with similar expression profiles and unrelated to metabolism. To this aim, we performed on the full RNA-seq dataset a weighted gene co-expression network analysis (WGCNA) [18], which classifies all co-expressed genes into modules (Fig. 7b). In this analysis, *Ass1* belonged to the *antiquewhite* module (1851 genes), whose regulation had the same positive direction in vWAT and scWAT at all time points. In contrast, *Nos2* belonged to the *Chocolate4* module that contained a total of 321 genes (Fig. 7c). Notably, at 8 weeks, the correlation of this module with the diet response was significant only in scWAT; while, at 20 weeks, genes in the module had an opposite regulation in vWAT and scWAT. To further explore the biological relevance of this set of co-expressed genes, we performed pathway enrichment analysis of all genes belonging to the *Chocolate4* module. The resulting GO terms were highly enriched for pathways linked to angiogenesis (Fig. 7d). Consistently, the expression levels of *Vegfa*, a potent angiogenic factor, as well as *Cdh5* and *Pecam1*, two endothelial cell markers, were strongly reduced in vWAT after 20 weeks of HFD, indicating a progressive impairment of angiogenesis in vWAT in response to the diet, as opposed to scWAT (Fig. 7e). Moreover, a number of the GO terms enriched in the *Chocolate4* module, including “Tissue migration”, “Epithelium migration” and “Epithelial cell migration”, were shared with the pathway analysis performed on cluster 1 of the ChIPseq analysis. Of note, several genes belonging to the Wnt pathway, such as *Ctnnbip1*, *Fzd8*, *Apc2*, *Ccdc88c*, *Wnt11*, *Tax1bp3*, and *Tmem88* were also listed in the *Chocolate4* module, which suggests again a key role of this signaling pathway in differentiating the responses of scWAT and vWAT to a HFD, not only at early time points, but also in the chronic setting.

## Discussion

**Fig. 8:**
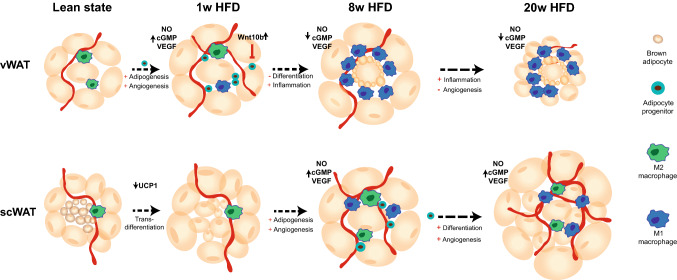
Differential response to HFD in vWAT and scWAT. Schematic illustration of the response to HFD feeding in vWAT and scWAT after 1, 8 and 20 weeks

Adipose tissue (AT) expansion upon over-nutrition has traditionally been associated mainly with adipocyte enlargement in response to the need of storing excess fat. However, as demonstrated here and in line with Jefferey et al. [1, 14], AT growth is tightly regulated by distinct molecular mechanisms within the different white adipose depots. In the early response to the diet (1 week), vWAT undergoes hyperplastic expansion with the commitment of adipocyte progenitors (APs), which form a pool of stem cells with adipogenic potential. Of note, we show that AP proliferation does not take place at this stage in scWAT, which instead shows early hypertrophic expansion (Fig. 8).

The presence of newly formed adipocytes should exert a beneficial effect on WAT health upon over-nutrition, since small adipocytes have been shown to counteract obesity-mediated metabolic decline and the onset of diabetes [23, 26, 49]. However, we also demonstrate that vWAT is subjected to anti-adipogenic signals that likely impair AP differentiation. Indeed, we observed reduced histone acetylation in the proximity of genes involved in fatty acid metabolism as well as in the regulation of cell growth and differentiation as early as 1 week, and further exacerbated at 8 weeks. Such a decrease in H3K27Ac implies reduced activity of the corresponding genes, suggesting decreased cell proliferation and differentiation. In particular, among the genomic regions with reduced H3K27Ac in vWAT, but no change in scWAT, we identified genes belonging to the canonical Wnt pathway, and to other fundamental pathways for cell differentiation (i.e. *Ihh, Bmp8a*). The increased gene expression of *Wnt10b*, a known anti-adipogenic mediator, together with the reduced phosphorylation levels of β-catenin uniquely in vWAT, further suggest a negative effect of HFD on AP differentiation in vWAT. One interesting feature of the genomic regions showing an opposing regulation of H3K27Ac and H3K4me1 in response to HFD is their lower acetylation and methylation, as well as their less abundant RNAPol II occupancy in scWAT compared to vWAT in control condition. Such an epigenetic landscape suggests that at these sites, critical for cell growth and differentiation regulation, the chromatin is more compacted and less prone to be transcribed in scWAT, possibly making this tissue less prone to hyperplastic expansion. Further studies will be necessary to understand the molecular basis underlying this different chromatin accessibility, which might be due to the activity of some tissue-specific transcription factor. Nevertheless, from a functional perspective, this hypothesis is in line with tracing experiments performed using the Adipochaser mice, showing that scWAT, when exposed to HFD, primarily undergoes hypertrophic expansion with a low rate of adipogenesis [48]. However, upon prolonged exposure to a HFD (8 weeks), and in contrast to vWAT, the acetylation level in these regions increases in scWAT, suggesting late activation of the adipogenic program in this depot. Accordingly, expression levels of cell cycle-related and histone genes, as well as *Zfp423* and *Fabp4,* are increased in scWAT at 8 and 20 weeks. Interestingly, measurements of BrdU incorporation in the AP pool after 8 weeks of diet, revealed a strong increase in the proliferation of these cells only in scWAT. This novel result proves the ability of scWAT to undergo hyperplastic growth upon prolonged exposure to overfeeding. This mechanism ensures the healthy expansion of the tissue and explains its lack of inflammation in diet-induced obesity. In contrast, the inhibition of adipocyte differentiation in vWAT could be the cause of the hypertrophic expansion of the tissue, due to mature adipocyte overload.

The importance of having a balance between hyperplastic and hypertrophic responses is due to the fact that the storage capacity of a fat cell is not unlimited. Once they reach their maximal size, adipocytes die [7]. This is consistent with the increased level of apoptotic genes observed in vWAT after 8 weeks of HFD (Fig. S6A). The concomitant appearance of immune cell recruitment in the tissue underscores the correlation between the increased cell death rate and the inflammatory response. Finally, it might also explain, at least in part, the remarkable decrease of vWAT weight after 20 weeks of HFD (Fig. 1b). Consistent with this, Strissel et al. [42] also observed an active process of remodeling after 16 weeks of HFD, in which the rate of adipocyte death (80%) exceeded the rate of tissue repair, resulting in net adipocyte and vWAT loss [42].

In contrast to vWAT, scWAT is known to undergo a metabolically healthy expansion upon overnutrition in both humans and mice, but the explanation for the distinct behavior of these tissues has not yet been elucidated. The hypotheses explaining this difference have generally been ascribed to different tissue origins, location and cell type. Our data add a new element to this scenario, by suggesting that the presence of both beige and white adipocytes is protecting scWAT from the onset of obesity-related inflammation. Indeed, the reduced number of beige adipocytes in scWAT during the early phase of overnutrition suggests that transdifferentiation of beige cells to white cells occurs, and that it might be fundamental in helping the storage of excessive fatty acids. Intriguingly, the presence of beige cells in scWAT is rather considered as protective against the development of diet-induced obesity because of their thermogenic capacity which favors energy expenditure [46, 50]. Therefore, the idea of pushing the differentiation of beige cells, also known as “browning” induction, has gained a lot of attention in the last decade as a therapeutic intervention against obesity [22, 29, 37]. Such “browning” process takes place, for instance, upon cold stimulation, which increases the number of beige adipocytes without any AP clonal expansion [6], suggesting that white adipocytes can trans-differentiate to beige adipocytes. Our data indicate that the opposite mechanism of transdifferentiation, from beige to white adipocytes, might also take place and, importantly, might have a positive outcome upon HFD, as it provides scWAT with new small mature adipocytes that can store fatty acid excess, although at the expense of the “burning” capacity of the tissue.

Our work is based on unbiased analyses of multiple genome-wide datasets that were obtained from vWAT and scWAT samples with a rigorously controlled study design. Besides applying classical pathway enrichment tools to our data, we further deepened our analyses by adapting emerging approaches that use modeling of cellular metabolism, such as metaboGSE [45] and genome-scale metabolic network (GSMN). Because of their totally different methodology, such novel tools enabled us to identify other key features that were not highlighted with classical pathway analyses and that could contribute to the different timing of the adipogenic program in vWAT and scWAT. In particular, the observation that important angiogenic genes, such as *Nos2, Cdh5, Pecam1* and *Vegfa*, have an opposing regulation in the two tissues in the late time points and increased expression in vWAT only at 1 week, could suggest a link between angiogenic and adipogenic programs. Further suggesting this possibility, our data indicate that HFD induces a tissue-dependent modulation of the Wnt pathway, which is known for its ability to finely modulate cell fate, angiogenesis and adipogenesis [4, 52]. However, more studies are necessary to dissect not only the molecular, but also the cellular players that are cross-talking in this connection. One possible scenario in this sense would be that reduced migration of epithelial cells, and thus reduced formation of new vessels, might affect the migration of adipocyte precursors, as previously reported [15, 31]. In addition, recent evidence highlighted the existence of a CD142+ adipogenesis-regulatory cell population in the stromal vascular fraction of adipose tissue [39]. These cells are localized in the proximity of blood vessels. A different activation of these cells in the vWAT and scWAT, respectively, might explain their different plasticity in response to HFD.

Altogether, comparing vWAT and scWAT responses to a HFD at different time points has been very successful in pinpointing molecular mechanisms potentially linked to initiation of inflammation in vWAT. In particular, despite an initial increase in the number of APs, vWAT undergoes a block in pre-adipocyte differentiation that prevents the formation of mature adipocytes. This then causes hypertrophic expansion of mature adipocytes that, after reaching their maximal size, undergo cell death and favor the recruitment of immune cells. Our data suggest that early intervention, facilitating differentiation of primed pre-adipocytes could be a strategy to help vWAT cope with the excess of fatty acids, reduce cell stress and death as well as improve the inflammatory response. The possible beneficial effect of favoring adipocyte differentiation in the treatment of obesity was in part already suggested by the positive outcome on insulin resistance of PPARγ agonists, which act, among others, by promoting adipogenesis. These drugs were discontinued due to several side effects associated to the multiple activities of PPARγ. Our results reinforce the idea of a key role played by adipocyte differentiation in the pathophysiology of the obese adipose tissue and emphasize the importance of understanding how this process is fine tuned in order to improve our chance to specifically target it.

## Methods

### Animal experiments

All animal experiments and procedures were approved by the Swiss Veterinary Office (VD-2942.b. and VD-3378). C57/BL6 male mice were purchased from Janvier Labs and housed 5 per cage in the animal facility of Centre for Integrative Genomics, University of Lausanne.

Four-week-old mice were fed for 2 weeks with a 10% in fat chow diet (D12450J, Research Diet). At 6 weeks of age they were either shifted to a high-fat diet (HFD) containing 60% fat (D12492, Research Diet) or kept on a control diet for 1, 8 and 20 weeks (Suppl Fig. S1A). Random blocking was used. Evolution of the diet-induced obesity was followed by regular measurements of weight (Fig. 1a). All animals were kept in a 12:12 h light:dark cycle with water and food ad libitum. All the mice were killed by CO_2_ between ZT2 and ZT5. For BrdU (Roche, 10280879001) treatment, BrdU was administered in the drinking water at 0.8 mg ml^−1^ and refreshed every two days.

In this study, vWAT refers to the perigonadal visceral adipose tissue in mice, while scWAT refers to inguinal subcutaneous adipose tissue in mice.

### Pooling strategy for ChIP-seq and RNA-seq

For ChIP-seq and RNA-seq experiment, a total of 170 and 180 mice were put on HFD and chow diets, respectively, to collect enough adipose tissue for chromatin preparation, which required pooling adipose tissue from different mice, as shown in Suppl. Fig. S1D. As the aim of the study was to identify changes responsible for the development of obesity-induced inflammation, we used the degree of vWAT inflammation as the pooling criteria for chromatin preparation from mice fed with a HFD for 8 and 20 weeks. In contrast, adipose tissue from mice fed with a chow diet (1, 8 and 20 weeks) and from mice fed a HFD for 1 week, where the treatment was too short to trigger vWAT inflammation, was arbitrarily pooled to prepare 2 chromatin samples per condition. The following parameters associated with vWAT inflammation were measured in all HFD fed mice (8 and 20 weeks), and in about 20 control mice (8 and 20 weeks): circulating levels of insulin, resistin and leptin levels, and expression of *Tnf, Ccl*-*2* and *Itgax* in vWAT. All these measurements, in addition to the individual mouse weight, were used as variables to perform a Principal Component Analysis (PCA). Low responders were identified as mice clustering close to the control group and with the absence of F4/80 positive cells measured by immunostaining (Figs. S6B, S6C) and were excluded for pooling.

For RNA-seq, RNA was extracted from about 30 mg of each individual adipose tissue. Equal amounts of RNA were pooled to obtain 6 independent replicates/conditions that were processed for RNA-seq. As depicted in Fig. S1D, each chromatin pool was paired with 3 RNA-seq pools (adipose tissue coming from the same mice).

### RNA sequencing

Subcutaneous and visceral adipose tissue was ground up in liquid nitrogen to perform RNA-seq analysis. The groups were as follows: (1) 1 week fed with control diet, (2) 1 week fed with HFD, (3) 8 weeks fed with control diet, (4) 8 weeks fed with HFD, (5) 20 weeks fed with control diet, (6) 20 weeks fed with HFD. For each group, 6 biological replicates were used, and each replicate was generated from a pool of mice (see Fig. S1D for details). RNA was extracted and purified using RNeasy Mini columns (Qiagen), checked for quality using the Bioanalyzer instrument (Agilent Technologies, Santa Clara, CA, USA) and subject to the NuGen RNA-seq kit (NuGen) for library preparation. Libraries were sequenced 8 for lane (total 8 lanes) on an Illumina HiSeq 2000 (at the Centre for Integrative Genomics, Lausanne) and around 20 or 30 million single-end reads were obtained per sample.

RNA-seq reads were subject to trimming of adapters and low-quality flanking ends using Cutadapt. Pre-processed reads were mapped to mm10 version GRCm38 using STAR aligner [9] and the estimation of isoform abundance was computed using RSEM [20]. Quality of the RNA-seq data alignment was assessed using RSeQC [47].

The list of RNAs was filtered to obtain those with a read coverage larger than 1 CPM (counts per million) in at least 1 sample, and 20,876 of the total 48,709 annotated RNAs survived this inclusion criterion. The edge R pipeline for differential expression analysis and the CPM calculation considered different library sizes and runs a normalization using TMM (trimmed mean of M-values algorithm) [34]. Unwanted variation due to epididymal tissue contamination was removed using the RUVr R package (v1.6.2) [32].

### Pathway analysis

Pathway analyses were performed with R using tools called Signaling Pathway Impact Analysis (SPIA) R package (v3.3.3) [43], ClusterProfiler R package (v3.2.14) [51] or WGCNA R package (1.63) [53]. SPIA analysis was performed on the full RNA-seq dataset (16,709 protein coding genes) without any a priori selection for fold change. The program was provided with BH-adjusted *p* value and fold change from differential expression analysis of HFD versus CTR groups at different time points. The differentially expressed genes were defined with a BH-adjusted *p* value cut-off lower than 0.05. ClusterProfiler with Gene Ontology (GO) annotation was applied, when specified, on specific list of genes.

A weighted gene network was built for WGCNA analysis by setting the soft threshold power, to which co-expression similarity is raised to calculate adjacency, as 6. Hierarchical clustering was performed using the Dynamic Tree cut algorithm [17], which returned 71 proper modules with sizes ranging from 2918 to 39 genes. Modules with similar profiles (distance lower than 0.18) were merged to have all the co-expressed genes in each module.

### ChIP sequencing

ChIP-seq was performed on visceral WAT (vWAT) and subcutaneous WAT (scWAT) from 6 groups of mice. The groups were as follows: (1) 1 week fed with control diet, (2) 1 week fed with HFD, (3) 8 weeks fed with control diet, (4) 8 weeks fed with HFD, (5) 20 weeks fed with control diet, (6) 20 weeks fed with HFD. For each group, 2 biological replicates were used, and each replicate was generated from a pool of mice (see Fig. S1D for details).

Frozen adipose tissue was first reduced to powder and then cross-linked with 0,5% formaldehyde for 8 min at RT on a shaking platform and then stopped using 125 mM of glycine. Chromatin shearing was performed using 12 cycles 30’’ON/60’’ OFF, on a Bioruptor Pico (Diagenode). H3K4me1 (Abcam, ab8895, RRID:AB_306847, used 2 μg), H3K27Ac (Abcam, ab4729, RRID:AB_2118291, used 2 μg) and RNAPoll II (Santa-Cruz, SC-67318, RRID:AB_2167642, used 10 μg) were used for immunoprecipitation (IP). Library preparation was performed using the Diagenode kit (C05010013) according to the manufacturer’s instructions. 100-base single-ended tags were mapped to the mouse genome Ensemble GRCm38 (mm10) by the Illumina pipeline Casava 1.82 using Elandv2e. Tags containing no signal or more than 5 mismatches, and tags that were mapped outside chromosome sizes of mm10 were filtered out. IP quality control and strength measurement were performed using CHANCE on BED files [8].

#### ChIP-seq: significant bins identification

The ChiP-Cor tool [2] was used to analyze the correlation between 5′ and 3′ tags position, determine the average ChIP fragment length for each sample and shift the signal accordingly. The mm10 genome was divided into 500 nucleotides-long consecutive, non-overlapping bins to gather the ChIP signal. The bin values were scaled by the total tags for each sample and 30 pseudo-counts were added to stabilize the variance of low scores.

The log2-scaled bin values of each ChIP sample and of its corresponding INPUT sample were used to produce a MA plot and identify significant bins. Bins with an adjusted false discovery rate (Benjamini–Hochberg) *p* value below 0.05 were considered significant. To avoid bin border effect, the whole process was repeated with bins shifted by a half bin size (e.g., 250 bases). Only bins that were significant in both biological replicates of the same condition were retained.

The collection of bins presenting a significant difference between ChiP and INPUT in at least one sample was retained as the final set of bins of interest. Bins separated by less than 500 nt were then merged to define continuous regions. A total of 72,314 significant regions were retained. For each sample, the tag density of each region was collected and normalized by the region width and by the total number of tags of each sample. Illumina sequencing data for RNA and ChIP-seq are available at GEO as the GSE132885.

### Flow cytometry analysis of cell proliferation

Isolation of adipose tissue stromal cells and mature adipocytes was performed as described [5]. Briefly, adipose tissue was excised, minced, and digested in Krebs–ringer phosphate buffer (KRP) containing 3% BSA (Sigma, 10775835001) and 1 mg ml^−1^ Collagenase Type 2 (Worthington Biochemical; LS004176) for 60 min in a shaking water bath at 37 °C. The mixture was then filtered (200 μm), and cells were centrifuged at 150*g* for 8 min. Mature cells were recovered and washed in KRP containing 3% BSA. Pelleted stromal cells were washed in PBS containing 3% FCS. For BrdU analysis, cells were stained with the following antibodies: CD45 PE-Cy7 (eBioscience 25-0451-82, RRID:AB_2734986, used at 1:1000), CD31 PE (Invitrogen 12-0311-82, RRID:AB_465632, used at 1:1000), CD29 PE-CpCy5.5 Invitrogen 46-0291-82, RRID:AB_10670099, used at 1:200), CD34 FITC (eBioscience 11-0.341-85, RRID:AB_465022, used at 1:25) and Sca-1 Pacific Blue (BioLegend 108120, RRID:AB_493273, used at 1:500). Cells were washed, fixed and permeabilized using BD Cytofix/Cytoperm, BD Perm/Wash and BD Cytoperm plus (BD Biosciences) according to the manufacturer’s recommendations. Cells were then treated with DNase (BD Biosciences; 1 mg/ml) in PBS for 1 h in a 37 °C water bath and then washed in BD Perm/Wash. Cells were then stained with anti-BrdU antibody (APC, BD Biosciences, used at 1:500) in BD Perm/Wash overnight in the dark at 4 °C. Cells were then washed in BD Perm/Wash, resuspended in PBS/3%FCS and analyzed on a BD FACS Canto. Data analysis was performed using Flowjo software (BD Biosciences). Live cells were selected as negative for Vivid Aqua (Invitrogen L34966, used 1:200). APs were gated as CD45^−^, CD31^−^, CD29^+^, CD34^+^ and Sca1^+^cells. BrdU incorporation was then measured in these cells using cells from a mouse that was not exposed to BrdU treatment as a negative control. Changes in BrdU incorporation in HFD-treated mice were calculated compared to control mice for each dissection day.

### Isolation of adipocyte nuclei

Briefly, adipose tissue was minced into approximately 2–3 mm pieces and gently digested in Krebs–ringer phosphate buffer (KRP) containing 3% BSA (Sigma, 10775835001) and 1 mg ml^−1^ Collagenase Type 2 (Worthington Biochemical; LS004176) for 60 min in a shaking water bath at 37 °C. The mixture was then filtered through a 200-μm filter, and cells were centrifuged at 150*g* for 8 min. Mature cells were recovered and washed in KRP containing 3% BSA.

Isolation of intact adipocytes was verified by staining for the plasma membrane with Cell Mask Orange (Invitrogen, C10045) and nuclei with DAPI as described [5]. Adipocytes were lysed in 0.02% NP40 in KRP by vortexing 10–20 s and then sitting at RT5 min. Adipocyte nuclei were isolated by centrifugation at 2000*g* for 5 min. For BrdU analysis, nuclei were fixed, permeabilized, and treated with DNAse and stained for BrdU as described above for stromal cells.

### Immunofluorescence

Adipose tissue was fixed in a IHC zinc fixative (BD Pharmingen, 550523), for 24–48 h at RT, and then washed with PBS. Samples were then dehydrated in increasing concentrations of ethanol and embedded in paraffin wax. 4-μm sections of paraffin blocks were then deparaffinized and rehydrated, followed by blocking step in 1% normal goat serum. Sections were stained with hematoxylin and eosin or blocking and staining was performed in 2% BSA in PBS. Incubation with primary antibodies including rat anti-BrdU (Abcam ab6326, RRID:AB_2313786, used at 1:350), rabbit anti-F4/80 (Abcam ab6640, RRID:AB_1140040, used at 1:800), anti-UCP1 (Abcam ab10983, RRID:AB_2241462, used at 1:500), was performed overnight at 4 °C. The Secondary antibodies Alexa Fluor 568 (Invitrogen a11077, RRID:AB_141874) and Goat anti-rat HRP (Life Technologies 31470, RRID:AB_228356), were used at 1:100, and incubated with tissue for 1–2 h at room temperature. Slides were mounted with DAPI and imaged by confocal microscopyCell Size quantification.

For each sample under analysis, six pictures of the Haematoxylin and Eosin sections were taken randomly at a magnification of 10 ×. Adipocyte size was calculated on each picture using the Adiposoft software [10]. This automated program retrieves the number and the area of each counted cell. All the information per section was pooled together and a distribution of the cell size was obtained using the function histograms in R. The number of cells was counted in a range of sizes from zero to 2000 μm^2^. A summary box plot was produced showing the variability in cell number in the range 0–50 µm^2^. Density plot for cell size distribution analysis was performed using the R function Density.

### Western blotting

Whole cell lysates were extracted from adipose tissue using mPER lysing buffer (Thermo, 78501) with 1 × protease inhibitor (Thermo, 78426) and 1 × phosphatase inhibitor (Thermo, 78429) cocktails. The lysates were left 1 h at 4 °C on a rotating wheel and then sonicated 5 cycles 30″ ON/30″ OFF, using a Bioruptor Pico (Diagenode). Protein concentration was determined by Pierce BSA protein assay Kit (23227). 10–15 μg of lysates was applied to SDS-PAGE. Total OXPHOS cocktail (Abcam, ab110413, RRID:AB_2629281, used at 1:250), anti-βcatenin (Cell signaling, 9582, RRID:AB_823447, used 1:1000), anti-Phospho-βcatenin (Cell signaling, 9561, RRID:AB_331729, used 1:1000) and anti-Vinculin (Abcam, ab129002, RRID:AB_11144129, used at 1:1000) antibodies were used for western blot.

### Metabolic modeling

The investigated GSMN was the iMM1415 mouse model [41], with the growth reaction rewritten to better meet the metaboGSE requirements (available from Supplementary Data). Essentially, the list of metabolites required for growth was manually completed and the ATP hydrolysis that generated “ATP-energy for growth” was separated from the growth reactions to remove ADP and phosphate from the product side of the growth equation. During preliminary investigations, we have considered the published adipocyte-specific GSMNs [24, 25], which could account for lipogenesis, lipolysis, and burning processes. These tissue-specific GSMNs, however, failed to yield better results than the generic iMM1415. Indeed, extraction of condition-specific sub-models is at the core of the metaboGSE algorithm, for which using a generic model as input is more suitable.

#### Gene ontology (GO) annotation

The GO annotation was obtained from the org.Mm.e.g.db R package (v3.6.0). We performed an initial GO term enrichment on the set of 1110 genes from the studied genome-scale metabolic network (GSMN) versus the complete genome using topGO (v2.32.0) with *weight01* algorithm and *fisher* statistic. This produced 678 GO terms in the biological process category, which were associated with at least 3 and at most 50 genes in the GSMN and enriched with *p* value < 0.1. Each GO term corresponds to a gene set to study with metaboGSE.

#### Permutation test for the discrepancy of a gene set across many conditions

As described in Tran et al. [45], for each RNA-seq sample, a series of metabolic sub-networks was built using the expression-based ranking of genes and a comprehensive genome-scale metabolic network (GSMN). Each sub-network was evaluated by a ${\text{fitness}}$ score, which estimates its degree of alteration from the comprehensive GSMN. A fitness of 1 signifies a sub-network with the same viability as the comprehensive GSMN; whereas, a fitness of 0 indicates a totally degraded model.

For a given gene set *g*, e.g., a set of genes associated with a GO term, a depletion curve $D$ was computed by considering the fraction of genes remaining in *g* as a function of

$$x = {\text{fitness}}^{*} = \left( {1 - i/k} \right) \cdot {\text{fitness}},$$

where $i$ is the number of genes to remove to obtain the corresponding sub-model and $k$ the number of genes in the comprehensive model. The depletion curve for a given condition $U$ is defined as the average of the depletion curves in all replicates $u$ of $U$ (Fig. S5A). For two conditions $U$ and $V$, we computed the absolute area between the two depletion curves as:

$$\bar{A}_{U,V} = \mathop \smallint \limits_{0}^{1} \left| {\bar{D}_{U} \left( x \right) - \bar{D}_{V} \left( x \right)} \right|{\text{d}}x.$$

The discrepancy of *g* across multiple conditions is measured by the maximum area formed by depletion curves in every pair of conditions:

$$A^{*} = \mathop {\hbox{max} }\limits_{U,V} \bar{A}_{U,V} .$$

We performed a permutation test for the statistical significance of the discrepancy of *g* across conditions with the test statistic $E = A^{*}$. Resampling was performed by permuting replicates between conditions, while keeping unchanged the number of replicates in each condition. The resulting *p* value indicates whether the depletion evolution of *g* in one condition differs from at least one of the others:

$$p = \frac{{\mathop \sum \nolimits_{E} {\mathrm{I\!I}}\left( {E \ge E_{\text{obs}} } \right) + 1}}{E + 1},$$

where $E_{\text{obs}}$ is the observed of value of the test statistic, $E$ the resampling value, and $E$ the number of times of resampling. The + 1 terms accounts for the fact that the observed sampling was found in the resampling.

#### Post hoc permutation test for pairwise contrasts across multiple conditions

Permutation tests were also performed for each pair of conditions. The test statistic is the area formed by the two depletion curves $\bar{A}_{U,V}$. The resampling and calculation of *p* values were performed as for the multiple conditions described above.

For a contrast between two conditions $U$ and $V$, we introduced an additional measure of ${\text{AUC}}$ (area under the curve) difference:

$$\Delta_{U,V} = {\text{AUC}}_{U} {-} {\text{AUC}}_{V} = \mathop \smallint \limits_{0}^{1} \left( {\bar{D}_{U} \left( x \right) - \bar{D}_{V} \left( x \right)} \right){\text{d}}x.$$

This measure helped to reveal the behavior of *g* in $U$ in comparison with $V$. The overall enrichment result can be visualized in a clustered heatmap on ${\text{sign}}\left( \Delta \right)E$ of given gene sets against pairwise contrasts (as shown on supplementary Figs. S4A, S5B).

#### Identification of potential causal genes

Genes causing a descent in the depletion curve of *g* in each sample can be characterized with the descent angles and descent positions in the depletion curve. The descent angles measure the influence of those genes; whereas, the descent positions identified by ${\text{fitness}}^{*}$ measure the degradation status of the corresponding sub-network. To eliminate genes with minor causal effect, they can be subsequently filtered by these two features: ${\text{fitness}}^{*} > 0.1$, ${\text{descent angle}} > 45^\circ$ in at least one condition with more than a half of its replicates; ${\text{RSD}}\left( {\overline{\text{fitness}}^{*} } \right) > 0.2$ and ${\text{RSD}}\left( {\overline{\text{descent angle}} } \right) > 0.2$, where $\overline{\text{fitness}}^{*}$ denotes the average ${\text{fitness}}^{*}$ per condition, $\overline{\text{descent angle}}$ denotes the average ${\text{descent angle}}$ per condition, and ${\text{RSD}}\left( x \right) = \frac{{{\text{mean}}\left( x \right)}}{{{\text{standard deviation}}\left( x \right)}}$. It is noteworthy that the potential causal genes do not necessarily belong to *g*, as they may cause depletion of *g* through propagation in the metabolic network.

### Quantification and statistical analysis

All statistical analyses were performed in the R environment. Differential expression for RNA-seq analysis was computed with limma [33] by fitting samples into linear models for each time point and tissue. Log fold change (logFC), the *t* statistic and the adjusted *p* value computed by the Benjamini–Hochberg method were reported for each comparison. Gene expression levels are represented as mean ± SEM and were analyzed by Student’s *t* test (Prism 7.0).

### Electronic supplementary material

Below is the link to the electronic supplementary material.

Supplementary material 1 (PDF 8218 kb)

## Data Availability

RNA- and ChIP-seq data are accessible in GEO, accession number GSE132885.
