# Supplementary material for: Anti-adipogenic signals at the onset of obesity-related inflammation in white adipose tissue
Source: Cell Mol Life Sci. 2020 Mar 11;78(1):227–47. doi: 10.1007/s00018-020-03485-z (PMC7867564; doi:10.1007/s00018-020-03485-z)
Supplement: Supplementary file 1 — Supplementary material 1 (PDF 8218 kb) [file 18_2020_3485_MOESM1_ESM.pdf]

Supplemental information

Anti-adipogenic signals at the onset of obesity–related inflammation in white adipose tissue

Tiziana Caputo[1], Van Du T Tran[2], Nasim Bararpour[3,4], Carine Winkler[1], Gabriela Aguileta[1], Khanh Bao Trang[1], Greta MP Giordano Attianese[1], Anne Wilson[5], Aurelien Thomas[3,4], Marco Pagni[2], Nicolas Guex[2,6], Béatrice Desvergne[1]* and Federica Gilardi[1,3,4]*.

[1] Center for Integrative Genomics, University of Lausanne, Lausanne, Switzerland

[2] Vital-IT Group, SIB Swiss Institute of Bioinformatics, Lausanne, Switzerland.

[3]Unit of Forensic Toxicology and Chemistry, CURML, Lausanne University Hospital-Geneva University Hospitals, Switzerland.

[4]Faculty Unit of Toxicology, CURML, Lausanne University Hospital, Faculty of Biology and Medicine, University of Lausanne, Switzerland.

[5]Department of Oncology, University of Lausanne, Epalinges, Switzerland.

[6]Bioinformatics Competence Center, University of Lausanne, Switzerland.

***Corresponding Authors:**

Federica Gilardi: federica.gilardi@chuv.ch

Béatrice Desvergne: beatrice.desvergne@unil.ch

Figure S1

(**A, B, C**) Schematic representation of the CTR and HFD feeding experimental procedure and of the BrdU administration protocol. (**D**) Number of mice used in each individual data point and pool for both ChIP-seq and RNA-seq. Low responders correspond to mice that were not included in the analysis because of their low inflammatory response after HFD treatment.

Figure S2

(**A**) SPIA plot performed in vWAT and scWAT at 20 weeks. The X-axis shows the over-representation evidence (P_{NDE}), while the Y-axis shows the perturbation evidence (P_{TERT}). Each pathway is represented by a dot. Pathways above the oblique red line (red dots) are significant at 5% after Bonferroni correction, while those above the oblique blue line are significant at 5% after FDR correction (blue dots). The vertical and horizontal thresholds represent the same corrections for the two types of evidence considered individually. Pathways with a log(P_{NDE}) higher than 15 are represented by a small vertical bar.

(**B**) Log2 Gene expression level of proliferation related genes *Mki67, E2f1* and *Zfp423* in vWAT (top) and scWAT (bottom) from controls or HFD treated mice after 1, 8 and 20 weeks. (n=6).

(**C**) Log2 Gene expression level of Mature adipocyte markers *Pparγ* in vWAT and scWAT from control or HFD treated mice after 1, 8 and 20 weeks. Fold change (Δ) is reported for both tissues. Significance between the indicated groups was calculated using a two-tailed Student's *t*-test. *(p-value < 0.05), ** (p-value < 0.01), *** (p-value < 0.001), ****(p-value < 0.0001).

Figure S3

(**A**) Box plot of average tag density on the 711 regions in cluster 2 (green) for RNAPolII, H3K4me1 and H3K27Ac. (**B**) Log2 Gene expression level of scWAT specific Wnt genes: *Wnt2, Wnt10b* (top) and vWAT specific: *Wnt2b, Wnt5b, Wnt7b* (bottom), calculated in all control mice from all time points (n= 18). Significance between the indicated groups was calculated using a two-tailed Student's *t*-test. *(p-value < 0.05), ** (p-value < 0.01), *** (p-value < 0.001), ****(p-value < 0.0001).

Figure S4

(**A**) GO terms enriched (p-value < 0.05) in pathway analysis using a metabolic model for at least 3 among 4 comparisons either between tissues (vWAT and scWAT) or between diets (HFD and control). For tissues, at least one comparison has test statistic $E \geq 0.1$. For diets, at least one contrast has $E \geq 0.05$. In magenta, GO terms related to cGMP. In blue, those related to thermogenesis for tissue contrasts. In magenta, those concerning prostaglandin biosynthesis. Black *: p-value < 0.05 in at least three among 4 comparisons, grey *: p-value < 0.05 in less than three among 4 comparisons. (**B**) Potential causal genes with their effect for angiogenesis-related GO terms.

Figure S5

(**A**) Depletion curves of the gene set g for two conditions U, V with their replicates. (**B**) Overview of enriched GO terms for all pairwise comparisons. GO terms enriched with $E \geq 0.1$ in at least one contrast, E is set to 0 when p-value ≥ 0.05. In blue, pairs involving contrast vWAT: scWAT. #: 4 contrasts between diets (HFD and control), *: 4 contrasts between tissues (vWAT and scWAT).

Figure S6

(**A**) Log2 Gene expression level of Apoptosis markers *Casp3* and *Bcl2l11* in vWAT (top) and scWAT (bottom) from control or HFD treated mice after 1, 8 and 20 weeks (n=6). Data are represented as mean ± SD. Significance between the indicated groups was calculated using a two-tailed Student's *t*-test. *(p-value < 0.05), ** (p-value < 0.01), ****(p-value < 0.0001)

(**B**) Principal Component Analysis (PCA) performed using 7 variables: gene expression levels of *Ccl2*, *Itgax* and *Cxcl12*, plasma levels of insulin, resistin and leptin, and individual mouse weight in 61 mice treated for 8 weeks either with CTR (16) (black) or HFD (45) (red). Each point represents a mouse. (**C**) Same as in B in 58 mice treated for 20 weeks either with CTR (20) (black) or HFD (38) (red). A red panel was added to indicate the mice selected as "high responders".

Table S1

List of commonly regulated genes between HFD and CTR in the “Focal adhesion” and “ECM-receptor interaction” pathways in both vWAT and scWAT after 1 week of HFD feeding, with log2 fold change and Benjamini-Hochberg adjusted *p*-value.

Table S2

List of regulated genes between HFD and CTR in the “Alcoholism” and “Systemic lupus erythematosus” pathways in vWAT after 1 week of HFD feeding, with log2 fold change and Benjamini-Hochberg adjusted *p*-value. Genes highlighted in bold are common to the two pathways.

Table S3

List of regulated genes between HFD and CTR in the “Parkinson disease”, “Alzheimer disease” and “Huntington disease,” pathways in scWAT after 1 week of HFD feeding, with log2 fold change and Benjamini-Hochberg adjusted *p*-value. Genes highlighted in bold are common to the three pathways.

Table S4

(**A**) List of regulated genes between HFD and CTR in the “Cell cycle” pathway in scWAT after 8 weeks of HFD feeding, with log2 fold change Benjamini-Hochberg adjusted *p*-value. (**B**) List of genes in the “Systemic Lupus Erythematosus” pathway that are shared between the three time points (1, 8 and 20 weeks) in both vWAT and scWAT

Supplementary 1

A

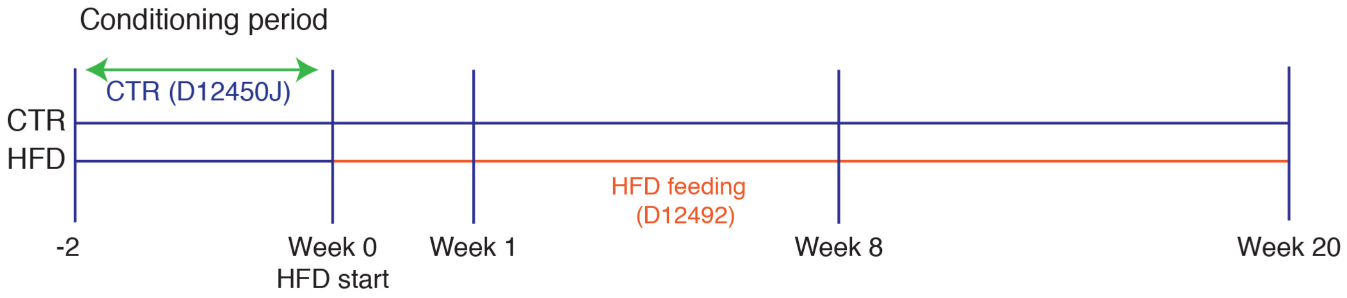

B

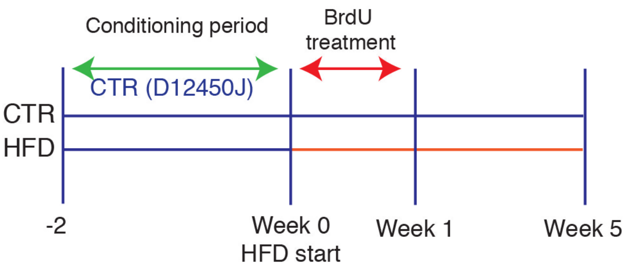

C

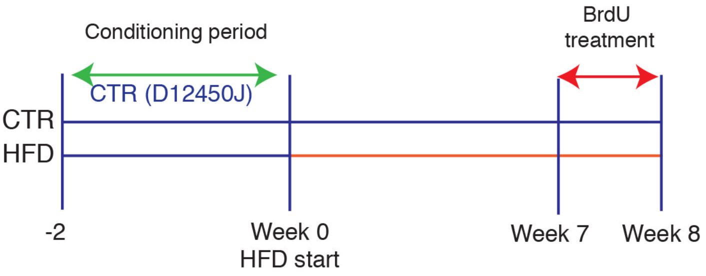

D

	**1 week**			
	CTR n=64		HFD n=48	
**ChIP-seq**	CTR1 n=32	CTR2 n=32	HFD1 n=24	HFD2 24
**RNA-seq**	CTR 1.1 n=10	CTR 2.1 n=10	HFD 1.1 n=8	HFD 2.1 n=8
	CTR 1.2 n=10	CTR 2.2 n=10	HFD 1.2 n=8	HFD 2.2 n=8
	CTR 1.3 n=10	CTR 2.3 n=10	HFD 1.3 n=8	HFD 2.3 n=8

	**8 weeks**				
	CTR n=40		HFD n=45		
**ChIP-seq**	CTR1 n=20	CTR2 n=20	HFD1 n=17	HFD2 n=17	"Low responders" n=11
**RNA-seq**	CTR 1.1 n=7	CTR 2.1 n=7	HFD 1.1 n=5	HFD 2.1 n=5	
	CTR 1.2 n=7	CTR 2.2 n=7	HFD 1.2 n=6	HFD 2.2 n=6	
	CTR 1.3 n=6	CTR 2.3 n=6	HFD 1.3 n=6	HFD 2.3 n=6	

	**20 weeks**				
	CTR n=26		HFD n=35		
**ChIP-seq**	CTR1 n=13	CTR2 n=13	HFD1 n=17	HFD2 n=17	"Low responders" n=1
**RNA-seq**	CTR 1.1 n=7	CTR 2.1 n=7	HFD 1.1 5	HFD 2.1 n=5	
	CTR 1.2 n=7	CTR 2.2 n=7	HFD 1.2 6	HFD 2.2 n=6	
	CTR 1.3 n=6	CTR 2.3 n=6	HFD 1.3 6	HFD 2.3 n=6	

Supplementary 2

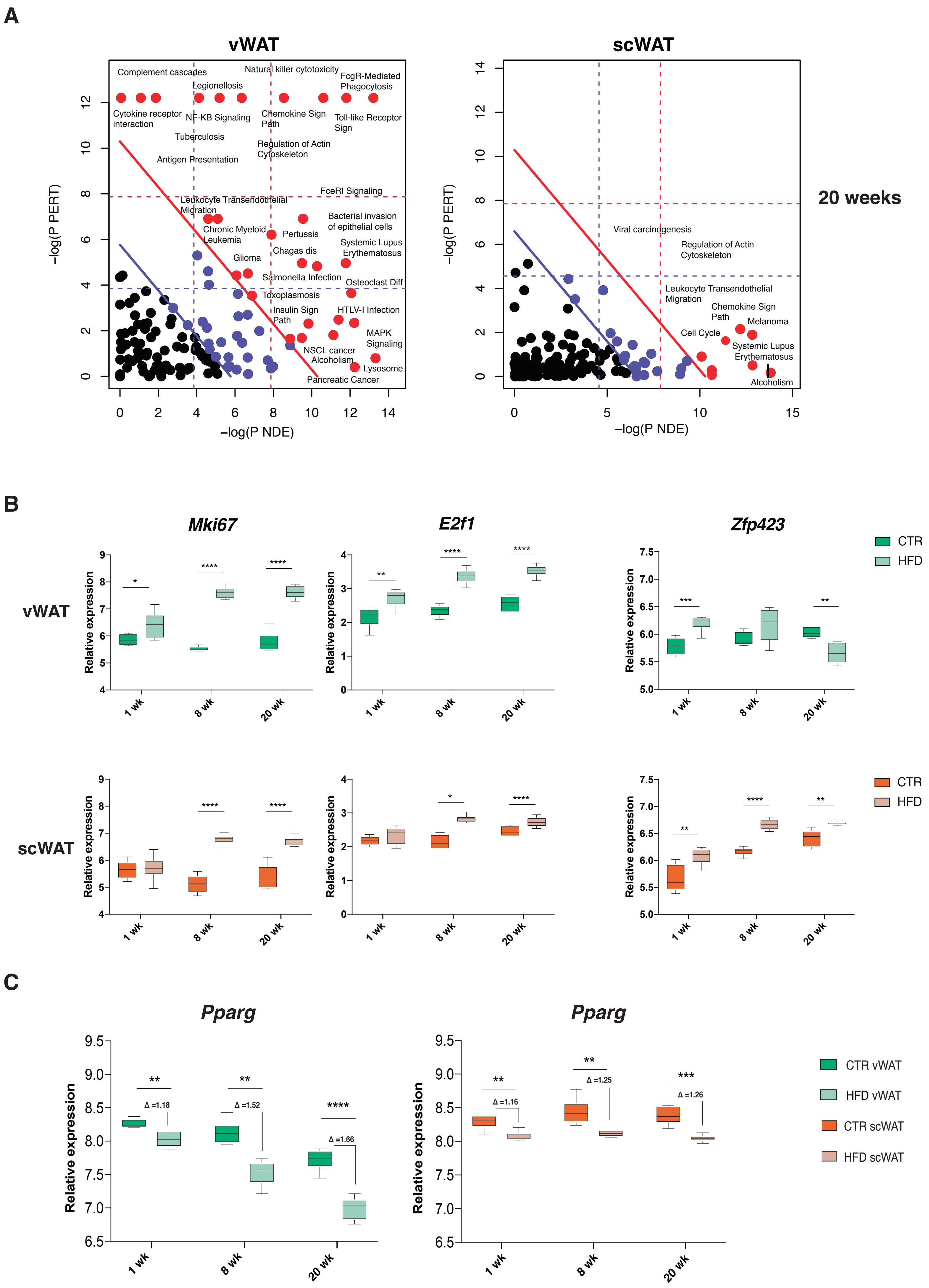

Supplementary 3

A

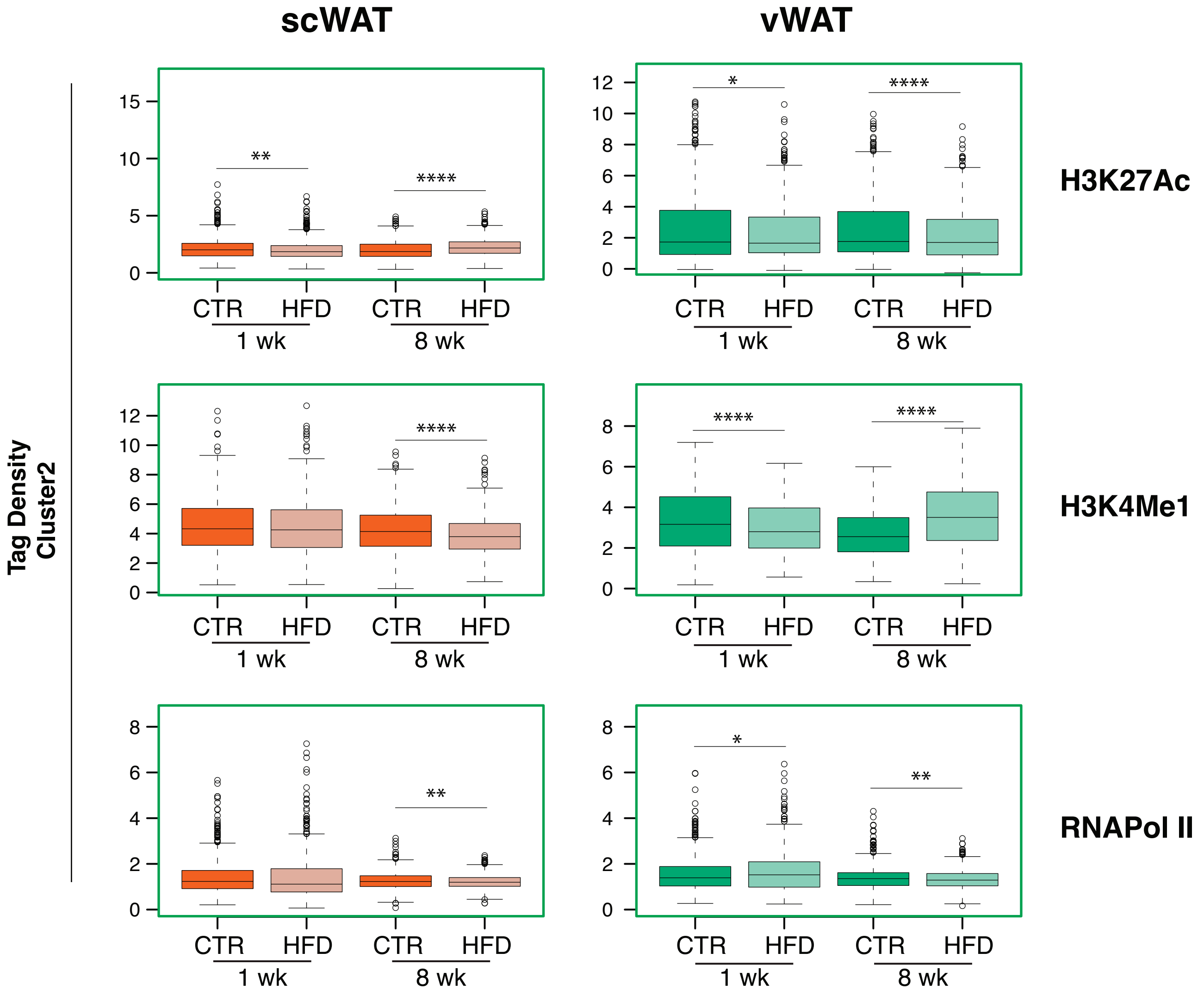

B

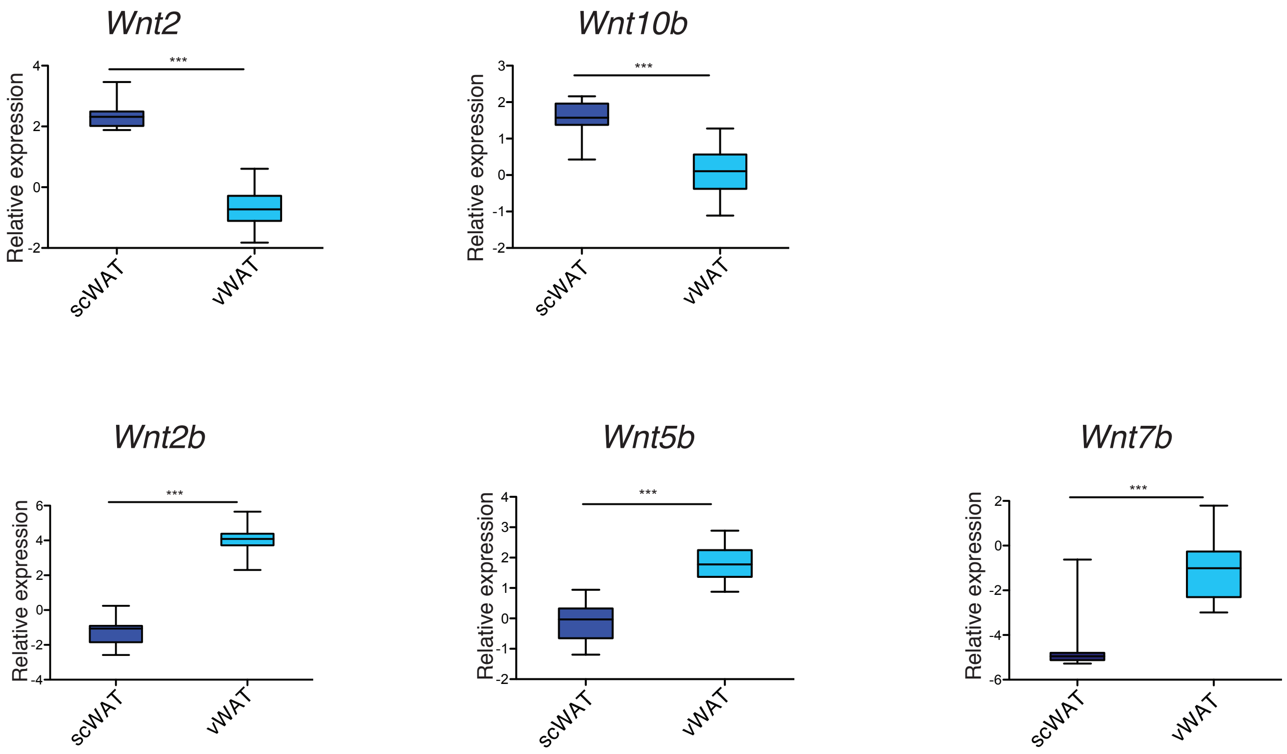

Supplementary 4

A

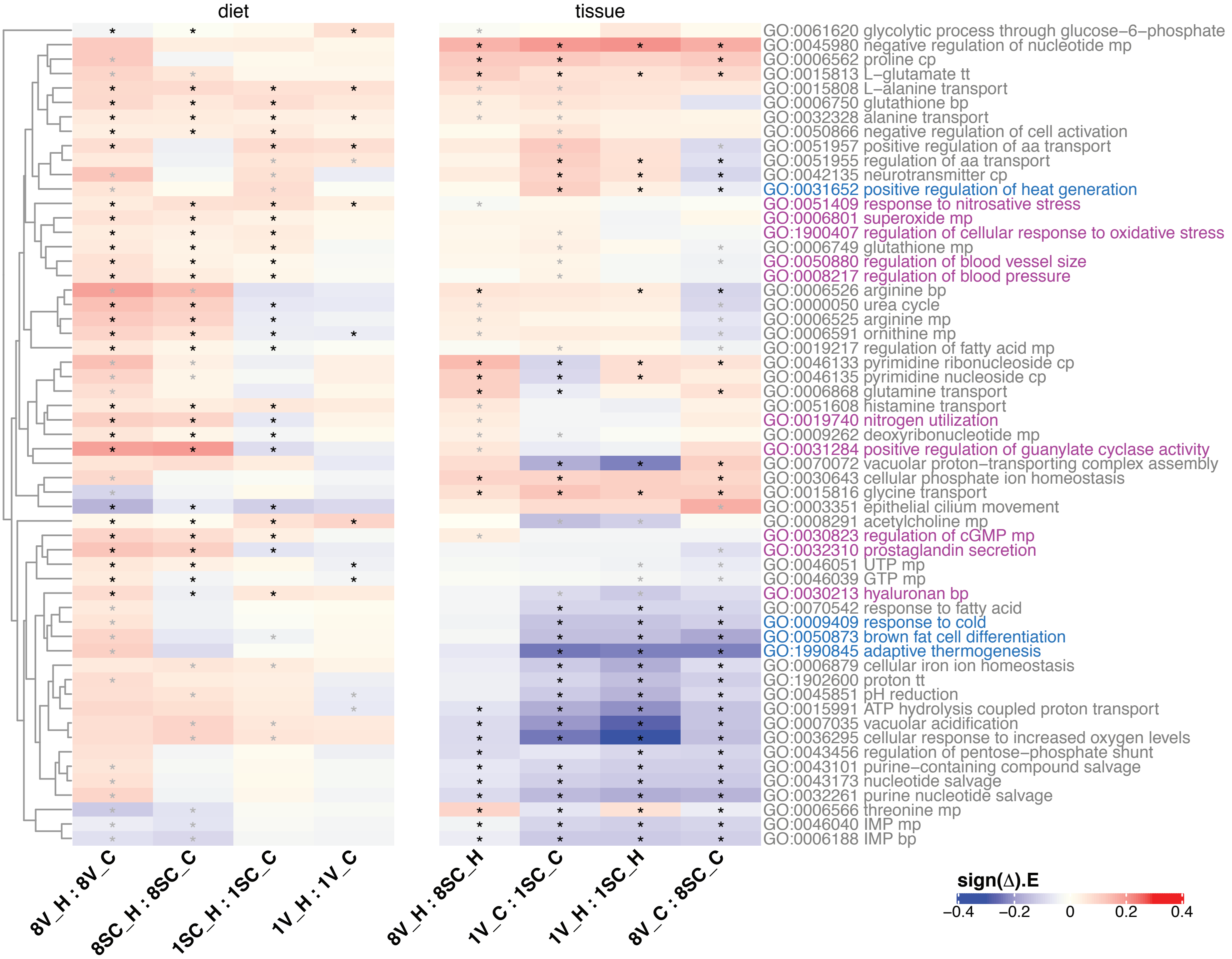

B

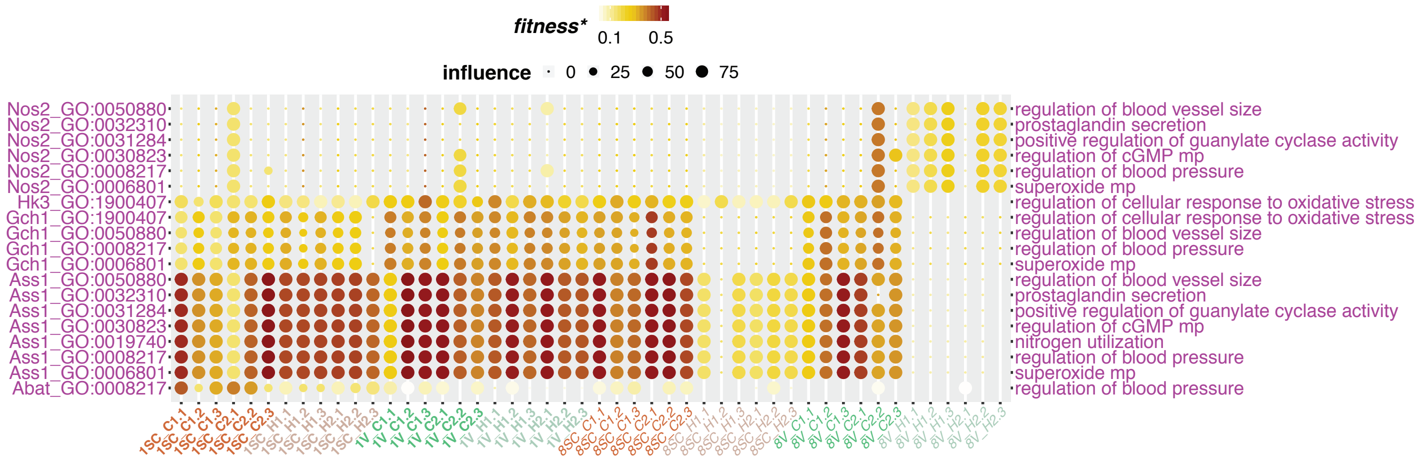

Supplementary 5

A

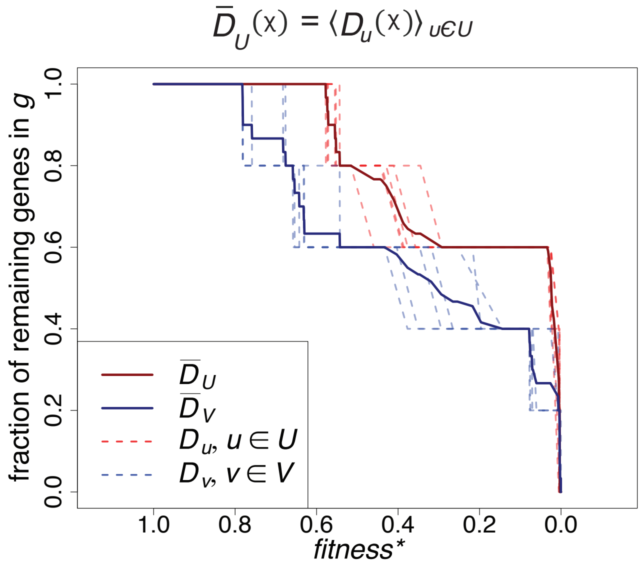

B

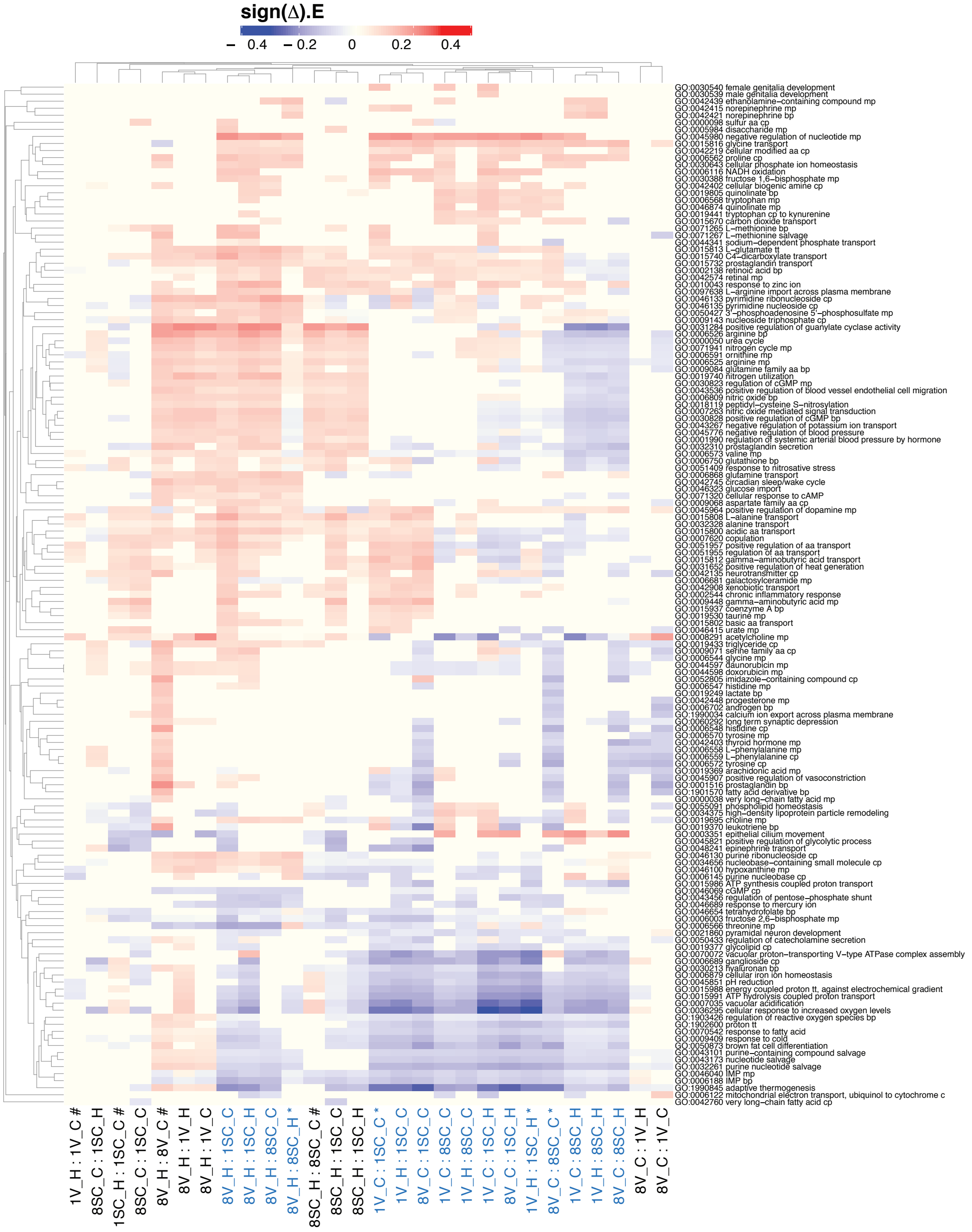

Supplementary 6

A

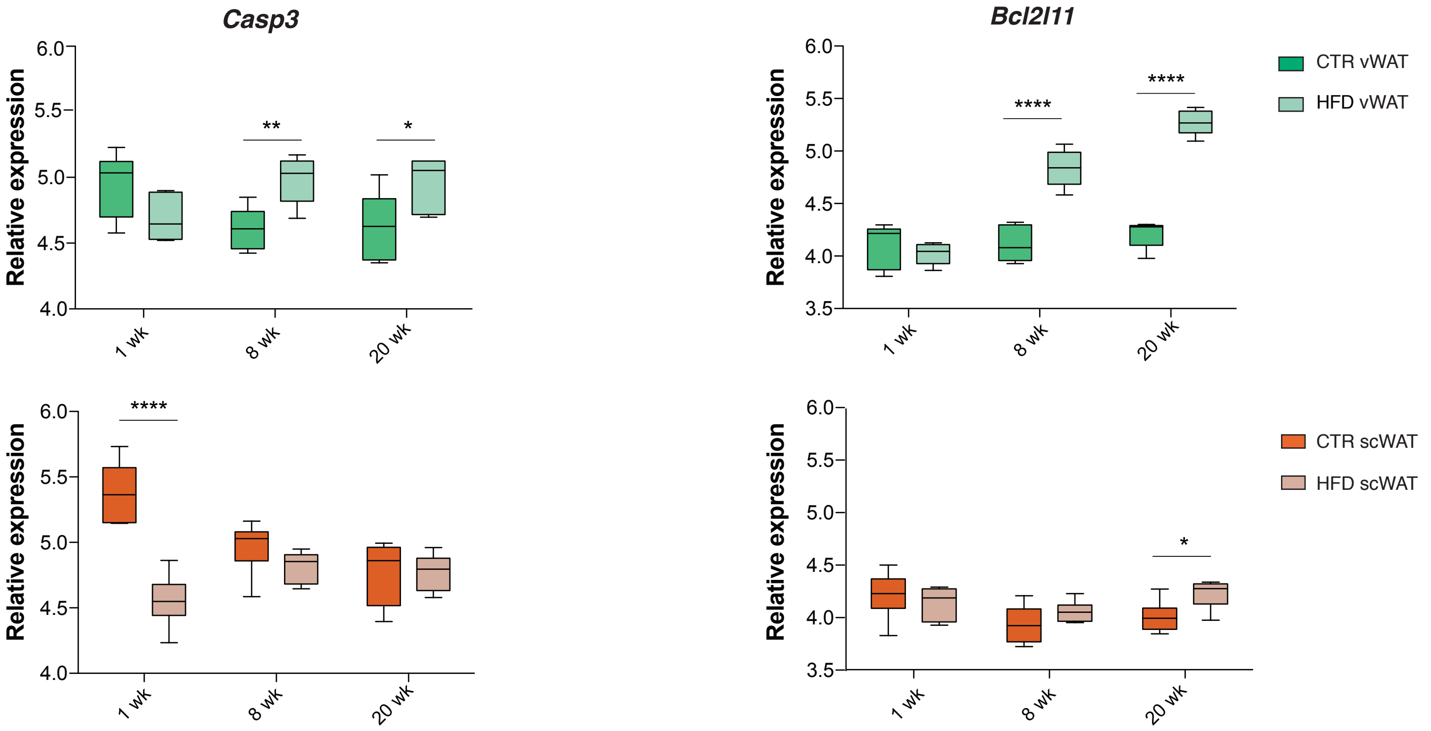

B

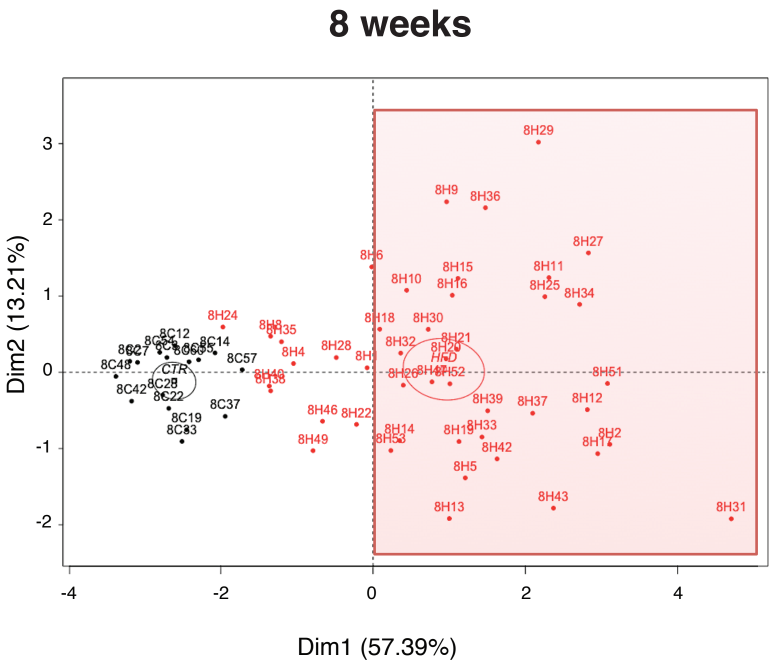

C

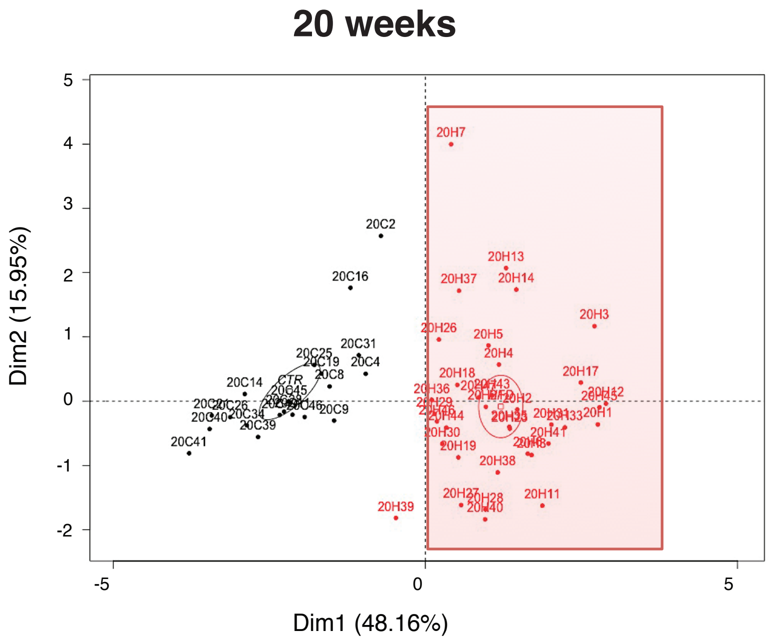

Table S1

Focal adhesion & ECM-receptor interaction
1 week

Gene Name	Fold Change vWAT	p. Value vWAT	Fold Change scWAT	p. Value scWAT
Akt1	0.30	1.3E-05	0.21	5.5E-04
Akt2	0.30	4.2E-05	0.23	7.7E-04
Capn2	0.25	2.2E-04	0.23	6.1E-04
Ccnd2	-0.22	6.1E-03	-0.33	1.3E-04
Col1a1	0.59	2.7E-04	0.63	1.2E-04
Col1a2	0.53	6.8E-05	0.64	6.2E-06
Col3a1	0.42	1.1E-04	0.58	1.7E-06
Col4a1	0.53	7.6E-06	0.45	6.8E-05
Col4a2	0.56	1.4E-05	0.42	3.3E-04
Col5a1	0.61	1.1E-07	0.58	2.4E-07
Col5a2	0.42	3.9E-06	0.48	6.1E-07
Col5a3	0.53	1.3E-04	0.35	6.0E-03
Col6a1	0.84	2.0E-10	0.68	7.7E-09
Col6a2	0.85	1.3E-09	0.59	5.3E-07
Col6a3	0.56	1.0E-06	0.44	3.4E-05
Flna	0.40	2.1E-06	0.29	1.1E-04
Flnb	0.40	1.2E-06	0.33	1.4E-05
Itga5	0.35	2.8E-03	0.33	4.4E-03
Itga7	0.61	6.3E-06	0.55	2.6E-05
Jun	0.44	2.0E-03	0.65	3.5E-05
Lamb2	0.48	1.7E-04	0.45	3.5E-04
Lamb3	0.63	1.8E-05	0.66	9.4E-06
Lamc1	0.24	3.1E-04	0.32	7.6E-06
Met	-0.30	7.9E-04	-0.27	2.3E-03
Myl12a	0.27	1.0E-04	0.30	3.6E-05
Parva	0.21	2.3E-03	0.18	5.9E-03
Pdgfa	0.37	2.0E-03	0.37	2.2E-03
Pdgfb	0.43	1.1E-04	0.31	2.6E-03
Thbs2	0.48	1.3E-03	0.60	1.5E-04
Thbs3	0.45	5.2E-04	0.60	1.6E-05
Tln1	0.34	7.1E-06	0.21	1.9E-03
Tln2	0.33	1.8E-03	0.53	1.0E-05
Tnxb	0.52	3.4E-04	0.55	1.9E-04
Vwf	0.45	6.5E-04	0.47	5.0E-04

Table S2

vWAT

Alcoholism 1 week

Gene Name	Fold Change	p.Value
Creb3l4	-1.39	0.00291
Hist2h2be	-0.36	0.00014
Hdac2	-0.24	0.00089
Atf2	-0.18	0.00334
Gnas	0.17	0.00039
Atf4	0.18	0.00373
Creb3	0.18	0.00038
Hist1h2be	0.26	0.00192
Calm3	0.27	0.00359
Hras	0.30	0.00251
Ntrk2	0.31	0.00002
Hdac5	0.31	0.00268
Adora2a	0.36	0.00191
Gnai2	0.37	0.00001
Gnb2	0.38	0.00070
Creb3l1	0.39	0.00224
Hist1h4d	0.41	0.00340
H2afj	0.42	0.00292
Hist2h4	0.54	0.00001
Hist1h4h	0.54	0.00035
Hist4h4	0.64	0.00157
Hist2h2ac	0.69	0.00104
Hist1h3e	0.74	0.00052
Hist1h3f	0.84	0.00016
Hist1h2bb	0.84	0.00017
Hist1h3a	0.87	0.00325
Hist1h2bg	0.87	0.00035
Hist2h2bb	0.92	0.00008
Hist1h3d	0.97	0.00127
Hist1h3b	0.97	0.00385
Hist1h3c	1.02	0.00027
Hist1h4f	1.04	0.00001
Hist1h3g	1.05	0.00266
Adcy5	1.06	0.00000
Hist1h2bh	1.08	0.00142
Hist1h2bn	1.11	0.00006
Hist1h2bl	1.17	0.00107
Hist1h2ab	1.19	0.00147
Hist1h2bj	1.19	0.00157
Hist1h2bk	1.20	0.00322
Hist1h2af	1.35	0.00027
Hist1h3i	1.36	0.00055
Hist1h2an	1.38	0.00329

Systemic Lupus Erythematosus 1, 8 & 20 weeks

Gene Name	Fold Change	p.Value
Hist2h2be	-0.36	0.00014
Hist1h2be	0.26	0.00192
Snrpb	0.31	0.00359
C4b	0.35	0.00525
C1qb	0.38	0.00065
Hist1h4d	0.41	0.00340
C1qa	0.42	0.00057
H2afj	0.42	0.00292
C1qc	0.44	0.00012
Hist2h4	0.54	0.00001
Hist1h4h	0.54	0.00035
Hist4h4	0.64	0.00157
Hist2h2ac	0.69	0.00104
Hist1h3e	0.74	0.00052
Hist1h3f	0.84	0.00016
Hist1h2bb	0.84	0.00017
Hist1h3a	0.87	0.00325
Hist1h2bg	0.87	0.00035
Hist2h2bb	0.92	0.00008
Hist1h3d	0.97	0.00127
Hist1h3b	0.97	0.00385
Hist1h3c	1.02	0.00027
Hist1h4f	1.04	0.00001
Hist1h3g	1.05	0.00266
Hist1h2bh	1.08	0.00142
Hist1h2bn	1.11	0.00006
Hist1h2bl	1.17	0.00107
Hist1h2ab	1.19	0.00147
Hist1h2bj	1.19	0.00157
Hist1h2bk	1.20	0.00322
Hist1h2af	1.35	0.00027
Hist1h3i	1.36	0.00055
Hist1h2an	1.38	0.00329

Table S3

1 week scWAT

Parkinson Dis

Gene Name	Fold Change	p.Value
Casp3	-0.82	8E-07
Ndufs4	-0.43	1E-06
Ubb	0.44	1E-05
Ndufb9	-0.37	3E-05
Sdhd	-0.37	3E-05
Cox5a	-0.47	4E-05
Ndufs1	-0.38	7E-05
Sdha	-0.30	1E-04
Uqcrh	-0.30	1E-04
Ndufv2	-0.38	2E-04
Ndufb5	-0.30	2E-04
Uqcrc2	-0.36	2E-04
Pink1	0.36	2E-04
Ndufa10	-0.37	3E-04
Uqcrq	-0.45	3E-04
Cycs	-0.66	3E-04
Atp5o	-0.24	7E-04
Cox7b	-0.40	8E-04
Ndufa6	-0.30	8E-04
Ndufa4	-0.32	9E-04
Cox7a2		E-04
		E-03
		E-03
Cox6b1	-0.32	1E-03
Sdhc	-0.24	2E-03
Ndufab1	-0.29	2E-03
Ndufa12	-0.34	2E-03
Lrrk2	-0.27	2E-03
Cox6c	-0.36	3E-03
Ndufa1	-0.30	3E-03
Ppif	-0.34	3E-03
Cox6a1	-0.31	4E-03
Vdac2	-0.20	4E-03
Ndufb6	-0.37	4E-03
Atp5a1	-0.22	4E-03
Uqcrb	-0.46	5E-03
Ndufa5	-0.32	5E-03
Sdhb	-0.23	5E-03

Alzheimer Dis

Gene Name	Fold Change	p.Value
Casp3	-0.82	8E-07
Cycs	-0.66	3E-04
Cox5a	-0.47	4E-05
Uqcrb	-0.46	5E-03
Uqcrq	-0.45	3E-04
Ndufs4	-0.43	1E-06
Cox7b	-0.40	8E-04
Uqcr10	-0.40	1E-03
Ndufs1	-0.38	7E-05
Ndufv2	-0.38	2E-04
Ndufb6	-0.37	4E-03
Ndufb9	-0.37	3E-05
Ndufa10	-0.37	3E-04
Sdhd	-0.37	3E-05
Uqcrc2	-0.36	2E-04
Cox6c	-0.36	3E-03
Atp5f1	-0.35	1E-03
Ide	-0.34	3E-03
Ndufa12	-0.34	2E-03
Ndufa4	-0.32	9E-04
Cox7a2	-0.32	9E-04
Cox6b1	-0.32	1E-03
Ndufa5	-0.32	5E-03
Cox6a1	-0.31	4E-03
Sdha	-0.30	1E-04
Uqcrh	-0.30	1E-04
Ndufb5	-0.30	2E-04
Ndufa6	-0.30	8E-04
Ndufa1	-0.30	3E-03
Ndufab1	-0.29	2E-03
Atp5o	-0.24	7E-04
Sdhc	-0.24	2E-03
Sdhb	-0.23	5E-03
Atp5a1	-0.22	4E-03
Capn2	0.23	6E-04
Calm3	0.29	2E-03
Psen2	0.36	1E-05
Apbb1	0.37	9E-04
App	0.41	9E-07
Lrp1	0.48	5E-05
Apoe	0.48	2E-05

Huntigton Dis

Gene Name	Fold Change	p.Value
Casp3	-0.82	8E-07
Cycs	-0.66	3E-04
Cox5a	-0.47	4E-05
Uqcrb	-0.46	5E-03
Uqcrq	-0.45	3E-04
Ndufs4	-0.43	1E-06
Cox7b	-0.40	8E-04
Uqcr10	-0.40	1E-03
Ndufs1	-0.38	7E-05
Ndufv2	-0.38	2E-04
Ndufb6	-0.37	4E-03
Ndufb9	-0.37	3E-05
Ndufa10	-0.37	3E-04
Sdhd	-0.37	3E-05
Uqcrc2	-0.36	2E-04
Cox6c	-0.36	3E-03
Atp5f1	-0.35	1E-03
Ppif	-0.34	3E-03
Ndufa12	-0.34	2E-03
Ndufa4	-0.32	9E-04
Cox7a2	-0.32	9E-04
Cox6b1	-0.32	1E-03
Ndufa5	-0.32	5E-03
Cox6a1	-0.31	4E-03
Sdha	-0.30	1E-04
Uqcrh	-0.30	1E-04
Ndufb5	-0.30	2E-04
Ndufa6	-0.30	8E-04
Ndufa1	-0.30	3E-03
Ndufab1	-0.29	2E-03
Atp5o	-0.24	7E-04
Sdhc	-0.24	2E-03
Sdhb	-0.23	5E-03
Atp5a1	-0.22	4E-03
Pparg	-0.21	2E-03
Tfam	-0.21	6E-03
Vdac2	-0.20	4E-03
Creb3	0.16	1E-03
Hip1	0.27	2E-03
Cltb	0.34	2E-05
Creb3l1	0.44	8E-04
Dnah3	0.79	5E-03

Table S4

A

Cell cycle
8 & 20 weeks
scWAT

Gene Name	Fold Change	p.Value
Cdk1	1.87	4.99E-10
Chek2	0.79	5.87E-10
Ccna2	1.66	7.83E-10
Cdkn1a	1.11	1.98E-08
Ccne1	1.02	1.53E-07
Mad2l1	0.72	2.91E-07
Bub1b	1.13	4.16E-07
Cdkn2c	-0.70	5.14E-07
Gadd45b	1.30	5.59E-07
Dbf4	0.89	1.21E-06
Mcm6	0.56	1.22E-06
E2f1	0.72	2.24E-06
Ccnb1	1.57	2.52E-06
Ccnb2	1.54	3.88E-06
Ywhah	0.43	4.27E-06
Cdkn2b	0.76	7.38E-06
Mcm3	0.55	7.53E-06
Tfdp1	0.27	9.59E-06
Mcm5	0.90	1.11E-05
Bub1	1.71	1.16E-05
Rad21	-0.30	2.71E-05
Orc1	1.64	2.82E-05
Rbl2	-0.22	2.92E-05
Cdc20	1.54	3.75E-05
Ccnd1	0.82	4.56E-05
Cdkn1c	-0.76	5.30E-05
Prkdc	-0.31	7.10E-05
Cdc25c	2.03	8.13E-05
Ywhaz	0.26	1.29E-04
Ep300	-0.32	1.34E-04
Cdc45	0.44	1.47E-04
Mcm2	0.54	1.53E-04
Ttk	1.41	1.76E-04
E2f3	0.30	2.32E-04
Plk1	1.32	3.16E-04
Mdm2	0.21	6.62E-04
Ccne2	0.62	6.95E-04
Ywhab	0.29	1.43E-03
Ywhaq	0.29	1.59E-03
Cdkn1b	-0.28	1.64E-03
Tgfb3	-0.31	1.75E-03
Gsk3b	0.25	1.89E-03
Stag1	-0.22	2.30E-03
Cdc27	0.29	2.41E-03
Orc3	-0.23	2.90E-03
Mad1l1	0.39	3.22E-03
E2f5	-0.28	4.26E-03
Crebbp	-0.20	4.37E-03
Espl1	0.83	4.52E-03
Pcna	0.30	5.27E-03
Cdc7	0.31	5.66E-03
Cdc14b	-0.23	6.43E-03
Stag2	-0.20	1.10E-02
Cdk6	0.40	1.36E-02
Cul1	-0.14	1.44E-02
Cdc6	0.34	1.56E-02
Tgfb1	0.31	2.30E-02
Hdac1	-0.13	3.76E-02

B

Systemic Lupus Erythematosus
1, 8 & 20 weeks

Gene Name
Hist2h2be
Hist1h2be
C4b
Hist1h3e
Hist1h3f
Hist1h2bb
Hist1h3a
Hist1h2bg
Hist2h2bb
Hist1h3d
Hist1h3b
Hist1h3c
Hist1h4f
Hist1h3g
Hist1h2bh
Hist1h2bn
Hist1h2bl
Hist1h2ab
Hist1h2bj
Hist1h2bk
Hist1h2af
Hist1h3i
Hist1h2an
